# Long-Term Outcomes of a Comprehensive Mobile Smoking Cessation Program With Nicotine Replacement Therapy in Adult Smokers: Pilot Randomized Controlled Trial

**DOI:** 10.2196/48157

**Published:** 2023-09-18

**Authors:** Jennifer D Marler, Craig A Fujii, MacKenzie T Utley, Daniel J Balbierz, Joseph A Galanko, David S Utley

**Affiliations:** 1 Pivot Health Technologies, Inc San Carlos, CA United States; 2 Department of Pediatrics University of North Carolina Chapel Hill, NC United States

**Keywords:** smoking cessation, digital health, smartphone, digital sensor, carbon monoxide, breath sensor, biofeedback, mobile apps, health promotion, app, mobile phone

## Abstract

**Background:**

Increased smartphone ownership has led to the development of mobile smoking cessation programs. Although the related body of evidence, gathered through the conduct of randomized controlled trials (RCTs), has grown in quality and rigor, there is a need for longer-term data to assess associated smoking cessation durability.

**Objective:**

The primary aim was to compare smoking cessation outcomes at 52 weeks in adult smokers randomized to a mobile smoking cessation program, Pivot (intervention), versus QuitGuide (control). The secondary aims included comparison of other smoking-related behaviors, outcomes and participant feedback, and exploratory analyses of baseline factors associated with smoking cessation.

**Methods:**

In this remote pilot RCT, cigarette smokers in the United States were recruited on the web. Participants were offered 12 weeks of free nicotine replacement therapy (NRT). Data were self-reported via a web-based questionnaire with videoconference biovalidation in participants who reported 7-day point-prevalence abstinence (PPA). Outcomes focused on cessation rates with additional assessment of quit attempts, cigarettes per day (CPD), self-efficacy via the Smoking Abstinence Self-Efficacy Questionnaire, NRT use, and participant feedback. Cessation outcomes included self-reported 7- and 30-day PPA, abstinence from all tobacco products, and continuous abstinence. PPA and continuous abstinence were biovalidated using witnessed breath carbon monoxide samples. Exploratory post hoc regression analyses were performed to identify baseline variables associated with smoking cessation.

**Results:**

Participants comprised 188 smokers (n=94, 50% in the Pivot group and n=94, 50% in the QuitGuide group; mean age 46.4, SD 9.2 years; n=104, 55.3% women; n=128, 68.1% White individuals; mean CPD 17.6, SD 9.0). Several cessation rates were higher in the Pivot group (intention to treat): self-reported continuous abstinence was 20% (19/94) versus 9% (8/94; *P*=.03) for QuitGuide, biochemically confirmed abstinence was 31% (29/94) versus 18% (17/94; *P*=.04) for QuitGuide, and biochemically confirmed continuous abstinence was 19% (18/94) versus 9% (8/94; *P*=.046) for QuitGuide. More Pivot participants (93/94, 99% vs 80/94, 85% in the QuitGuide group; *P*<.001) placed NRT orders (mean 3.3, SD 2.0 vs 1.8, SD 1.6 for QuitGuide; *P*<.001). Pivot participants had increased self-efficacy via the Smoking Abstinence Self-Efficacy Questionnaire (mean point increase 3.2, SD 7.8, *P*<.001 vs 1.0, SD 8.5, *P*=.26 for QuitGuide). QuitGuide participants made more mean quit attempts (7.0, SD 6.3 for Pivot vs 9.5, SD 7.5 for QuitGuide; *P*=.01). Among those who did not achieve abstinence, QuitGuide participants reported greater CPD reduction (mean −34.6%, SD 35.5% for Pivot vs −46.1%, SD 32.3% for QuitGuide; *P*=.04). Among those who reported abstinence, 90% (35/39) of Pivot participants and 90% (26/29) of QuitGuide participants indicated that their cessation program helped them quit.

**Conclusions:**

This pilot RCT supports the long-term effectiveness of the Pivot mobile smoking cessation program, with abstinence rates durable to 52 weeks.

**Trial Registration:**

ClinicalTrials.gov NCT04955639; https://clinicaltrials.gov/ct2/show/NCT04955639

## Introduction

### Background

In 2020, a total of 12.5% of adults in the United States (approximately 30.8 million people) smoked cigarettes [1]. Although this represents a marked decrease in the peak prevalence of 42.6% in 1964, smoking remains a formidable public health problem as the leading preventable cause of death, illness, and disability in the United States [2,3].

Behavioral interventions and pharmacotherapy have proven to be effective smoking cessation tools. Traditionally, behavioral intervention via counseling has been delivered in person in one-on-one or group sessions or remotely through phone quitlines. Food and Drug Administration (FDA)–approved pharmacotherapy such as nicotine replacement therapy (NRT) is available over the counter and by prescription; bupropion and varenicline are available by prescription. The use of combined behavioral support and pharmacotherapy increases the chance of smoking cessation by 70% to 100% compared with just brief advice or support [4]. The use of monotherapy is also effective, with NRT, bupropion, varenicline, and individual counseling increasing the chance of success by 50% to 60%, 50% to 80%, 100% to 140%, and 40% to 80%, respectively, compared with minimal support or placebo [4,5].

Unfortunately, the impact of these interventions is limited; less than one-third of adult cigarette smokers who make a quit attempt use any type of cessation counseling or FDA-approved pharmacotherapy [6]. Most quit attempts are unassisted, with resultant success rates of approximately 7% to 8% [3].

The rise of mobile smoking cessation interventions seeks to address this shortcoming by leveraging the ubiquitous nature of smartphones; in 2021, a total of 85% of adults in the United States owned a smartphone [7]. Assessment of earlier smartphone app–based smoking cessation programs has reported focus on ease-of-use features and simplistic tools, with low inclusion rates of evidence-based approaches such as the 5 A’s (Ask, Advise, Assess, Assist, and Arrange follow-up), counseling, and pharmacotherapy [8-10].

More recently, there has been both an increased presence of evidence-based cessation tools in mobile app–based smoking cessation programs and an increase in the rigor with which these programs are assessed, with the publication of several relevant randomized controlled trials (RCTs). BinDhim et al [11] compared an interactive decision aid app (intervention) with a static information app (control) for smoking cessation in an automated double-blind RCT. At 6 months, self-reported continuous abstinence was achieved in 7.3% (25/342) of participants in the intervention arm and 3.2% (11/342) in the control arm (intention to treat [ITT]; relative risk [RR]=2.27, 95% CI 1.09-4.86; *P*=.03) [11]. Garrison et al [12] compared mobile mindfulness training through the Craving to Quit app plus experience sampling (intervention) with experience sampling only (control) in a researcher-blind, parallel RCT. They reported biovalidated 7-day point-prevalence abstinence (PPA) in 9.8% (14/143; intervention) versus 12.1% (22/182; control) of patients (ITT; *χ^2^*_1_=0.4, *P*=.51) at 6 months [12]. Etter and Khazaal [13] conducted a 2-arm, parallel-group, individually randomized, double-blind RCT, in which the Stop-tabac app (intervention comprising an app with informational text, personal calculators, ecological momentary intervention for challenging situations, a quiz, contact information for telephone quitlines, coaching via automated messages, a discussion forum, and modules focused on NRT and electronic cigarettes) was compared with a control app (5 brief information pages and the aforementioned calculators). At 6 months, 7-day PPA was reported in 11.29% (298/2639) of intervention arm participants and 11.98% (318/2654) of control arm participants (ITT; odds ratio [OR] 0.94, 95% CI 0.79-1.11; *P*=.43) [13]. Finally, Bricker et al [14] compared iCanQuit (intervention; an acceptance and commitment therapy–based app) with QuitGuide (control; a United States Clinical Practice Guideline [USCPG]–based app from the National Cancer Institute [NCI]) in a 2-group, stratified, double-blind, individually randomized clinical trial in smokers. At 6 months, 29.57% (359/1214) of participants in the intervention arm and 21.57% (259/1201) of participants in the control arm self-reported 7-day PPA (ITT; OR 1.62, 95% CI 1.34-1.95; *P*<.001) [14].

Although the aforementioned RCTs evaluated behavioral intervention–focused mobile smoking cessation programs, a few RCTs have assessed similar mobile interventions with an enhanced evidence-based component, specifically, the provision of NRT. Hébert et al [15] conducted a 3-armed pilot RCT in which participants were randomized to Smart-T2 (a “just-in-time adaptive intervention that uses ecological momentary assessments to assess the risk for imminent smoking lapse and tailor treatment messages based on the risk of lapse and reported symptoms”), the NCI QuitGuide app, or usual tobacco cessation clinic care. Participants received 2 weeks of NRT. At 12 weeks, biovalidated 7-day PPA was reported in 22% (6/27) of Smart-T2 participants, 15% (4/27) of QuitGuide participants, and 15% (4/27) of usual care participants (ITT; *P*>.05) [15]. Webb et al [16] led a 2-arm, single-blinded, parallel-group RCT; participants were randomized to Quit Genius (intervention; a smartphone app delivering cognitive behavioral therapy content, one-to-one coaching, craving tools, and tracking capabilities) or very brief advice (control; in-person advice informed by the Ask, Advise, and Act model). Participants had the option to receive 12 weeks of NRT. Biovalidation via carbon monoxide (CO) breath sampling was performed in slightly less than half of the participants. At 6 months, 35.9% (95/265) of Quit Genius participants and 27.6% (73/265) of very brief advice participants achieved 7-day PPA (ITT; RR=1.32, 95% CI 1.03-1.69; *P*=.03) [16].

Similarly, we performed a remote pilot RCT in which participants were randomized to Pivot (intervention; an app-based smoking cessation program comprising activities and lessons, a personal CO breath sensor, in-app text-based human-provided coaching, NRT, and a moderated web-based community) or the NCI QuitGuide app (control). Participants had access to 12 weeks of NRT. At 6 months, 36% (34/94) of Pivot participants and 27% (25/94) of QuitGuide participants self-reported 7-day PPA (ITT; RR=1.3, 95% CI 0.8-1.9; *P*=.27), and 28% (26/94) of Pivot participants and 15% (14/94) of QuitGuide participants achieved biovalidated abstinence (ITT; RR=1.9, 95% CI 1.1-3.5; *P*=.02) [17]. In the context of these RCTs, the natural next step in the evolution of data pertaining to mobile smoking cessation programs is the establishment of longer-term outcomes through smoking cessation durability.

### Objectives

The primary aim of this study was to assess longer-term smoking cessation outcomes in the aforementioned RCT, in which Pivot was compared with QuitGuide [17]. Specifically, we aimed to compare self-reported and biovalidated PPA and continuous abstinence at 52 weeks after enrollment. Secondary aims included the assessment of other aspects of smoking-related behavior, such as quit attempts, cigarettes per day (CPD), and self-efficacy, and the performance of exploratory analyses of predictors of smoking cessation.

## Methods

### Design

In this 2-arm, parallel-group, noncrossover, single-center RCT, participants were randomized to 1 of 2 app-based smoking cessation programs: Pivot (intervention) or QuitGuide (control). All participants had access to 12 weeks of free NRT. The 6-month outcomes, including comparison of user engagement and retention, attitudes toward quitting, smoking behavior, and participant feedback, have been previously reported [17]. In this paper, we report the outcomes at 52 weeks. This study was performed remotely on an ambulatory basis. All participants provided electronic informed consent before taking part.

### Participants

Eligibility criteria were being aged ≥21 years, being a daily cigarette smoker (≥5 CPD) for the previous 12 months, having plans to quit smoking in the next 30 days, being a resident of the United States, being able to read and comprehend English, owning and using a smartphone compatible with the study app (iPhone 5 and above with operating system iOS 12 and above or Android 7.0 and above with operating system Android 7.0 and above), having daily internet access on smartphone, and self-reporting comfort with downloading and using smartphone apps.

Exclusion criteria were pregnancy (self-reported); health contraindications to NRT use (irregular heartbeat, high blood pressure not controlled with medication, myocardial infarction or stroke within the last 2 months, breastfeeding, skin allergies to adhesive tape or serious skin problems, stomach ulcers, and history of seizures); use of other smoking cessation support, including apps or actively taking medication to quit smoking; daily marijuana use; residence with another study participant; an immediate family member being a study participant; failure to provide contact information or verify email address; and participation in a previous study sponsored by Pivot Health Technologies Inc (formerly Carrot Inc).

### Recruitment

Participants were recruited in the United States through web media (Facebook and Google Ads). Potential participants were asked to provide contact information and answer questions on demographics using a web-based screening form. Study staff reviewed each web-based screening form.

Using nonproportional quota sampling, potential participants were called on a first-come, first-served basis, with the aim of enrolling 40% to 60% men, no more than 50% of participants from any decade-spanning age group (eg, 30-39 years), no more than 70% of participants in the non-Hispanic White race category, and up to 20% of participants not employed. The goals of these nonproportional quota sampling ranges were to ensure representation among men, racial and ethnic minority groups, age groups, and individuals of varying socioeconomic status.

During the screening phone call, potential participants were asked questions to confirm study eligibility. During this call, study personnel informed the potential participants of the study details and answered any questions. Potential eligible participants who wanted to proceed with the study were emailed an electronic Health Insurance Portability and Accountability Act (HIPAA) authorization form and an electronic informed consent form, which they signed before participating in the study.

### Randomization and Blinding

Participants were randomly assigned in a computer-generated 1:1 ratio to either QuitGuide or Pivot using randomly permuted blocks of sizes 2 and 4. The allocation sequence was provided by the Study Randomizer software application (2017) [18]. Participants were stratified by daily smoking frequency (≤14 vs ≥15 CPD), employment status (full-time or part-time employment vs not employed), race (minority race vs White), and expected difficulty to stay quit (DTQ; scale of 1-10; self-reported score of ≤5 vs ≥6). These 4 factors were chosen as they have been associated with cessation outcomes in previous studies [19-25]. The researchers were blinded to treatment allocation until after randomization was performed.

### Intervention: Pivot

Pivot is a 12-month digital smoking cessation program based on the USCPG for tobacco cessation. Pivot includes the Pivot Breath Sensor and Pivot app (Pivot Health Technologies Inc).

The Pivot Breath Sensor is a portable, personal mobile breath sensor that measures the level of CO in the exhaled breath. The user submits a breath sample by exhaling into the sensor mouthpiece. The sensor displays the exhaled breath CO value in parts per million (ppm) to the user directly on the device. When paired with the user’s smartphone, the user’s CO values also populate the user’s Pivot app. Displayed CO values are color coded and categorized as most consistent with not smoking (green; 0-6 ppm), possibly smoking (orange; 7-9 ppm), or smoking (red; ≥10 ppm).

The self-guided Pivot app leverages evidence-based principles and clinical best practices. This includes the USCPG-recommended 5 A’s; tailoring on readiness to quit [4]; the provision of FDA-approved NRT with accompanying education on use and adherence [4,26,27]; the incorporation of effective methods for smoking cessation based on cognitive behavioral therapy and self-determination theory [3,28,29]; and cognitive behavioral therapy–based counseling through a live, dedicated coach [3,4,30]. Pivot app functions include interactive educational activities and the ability to log cigarettes, set a quit date, create a quit plan, complete practice quits (1-24 hours in duration), play educational games, watch educational videos, interact with one’s dedicated human coach via in-app SMS text messaging, view CO breath sample values and trends, learn about and then order NRT, access the moderated web-based Pivot community discussion forum, share goals and progress with the web-based Pivot community discussion forum or one’s social network via SMS text messaging or email, and complete daily check-ins after the quit date.

The educational journey in the Pivot app comprises 4 tracts—Learn, Reduce, Prepare to Quit, and Maintain My Quit—and is designed to accommodate smokers along the spectrum of readiness to quit. Participants may navigate between tracts as desired to access content most relevant to their goals and needs.

Pivot users are assigned a human coach with whom they work one-on-one over the duration of their use of Pivot (up to 1 year). Communication between the coach and Pivot user is via asynchronous in-app SMS text messaging. Pivot coaches are tobacco treatment specialists. The coach reaches out periodically, approximately once per week, during the participant’s active use of Pivot. Participants may reach out to their coach whenever and however often they like.

Pivot users may access the moderated web-based discussion community through the Pivot app. The forum is moderated by a tobacco treatment specialist. The web-based community forum is a place to give and receive support and advice from others going through the Pivot program.

### Control: QuitGuide

QuitGuide is a product of Smokefree [31], a smoking cessation resource created by the Tobacco Control Research Branch at the NCI in collaboration with tobacco control professionals and smoking cessation experts and with input from ex-smokers [32]. A well-established smoking cessation app, QuitGuide has been used in previous RCTs in which digital smoking cessation programs were compared [14,15,33]. The app focuses on helping users understand their smoking patterns and build the skills needed to become and stay smoke-free [32]. Specifically, QuitGuide helps users focus on motivations to quit; prepare to quit by developing a quit plan, identifying and planning how to address triggers and moods, teaching about FDA-approved smoking cessation medications, and identifying and providing access to social support; quit smoking by acknowledging user progress and teaching skills to address cravings; and stay quit by presenting tips and motivations to stay smoke-free and address slips if they occur. The QuitGuide app functions include educational reading activities, including focus on FDA-approved cessation medications and associated adherence. Additional QuitGuide app functions include tracking and reviewing cigarettes, moods, triggers, and cravings; setting tip message notifications for locations and times when one is prone to smoke; setting a quit date; creating a quit plan; completing journal entries; sharing goals and progress with one’s social network via SMS text messaging or email; accessing additional chat and phone support; and providing updates on quit status after the quit date.

QuitGuide was used as the control for the following reasons: the content follows the USCPG for tobacco cessation; it is an app-based smoking cessation program, thereby enabling intrastudy comparison of same-modality interventions; the app is nonproprietary and free to the public; and its use in previous well-designed RCTs [14,15,33] provides context and enables interstudy comparison with earlier data.

### NRT Provision

Participants had access to free, FDA-cleared, over-the-counter NRT. Participants were provided with on-label information about the NRT and were able to order it on the web (QuitGuide) or in their study app (Pivot). The types of NRT offered included nicotine patches (7, 14, or 21 mg), nicotine gum (2 or 4 mg), and nicotine lozenges (2 or 4 mg). Participants could order patches, gum, or lozenges alone as monotherapy or patches with either gum or lozenges as combination therapy. Participants were able to order NRT every 2 weeks for up to a 12-week course over the first 12 months of the study.

### Biovalidation

Biovalidation was sought at 52 weeks in individuals who reported 7-day (or greater) PPA on the associated questionnaire. A video call with study staff and the participant was scheduled within 7 days following the participant’s response to the associated questionnaire. At the beginning of the biovalidation visit, participants were asked their CPD, 7-day PPA status, and whether they had smoked any other noncigarette (eg, pipes, cigars, or hookah) or combustible materials (eg, cloves or marijuana) over the previous 24 hours.

Participants who indicated that they were not at least 7 days abstinent or that they had smoked ≥1 CPD were not eligible to undergo further biovalidation testing during the visit. Participants who indicated that they were at least 7 days abstinent and had not smoked cigarettes were eligible to proceed with the testing. Participants who indicated that they had smoked any other combustible materials over the previous 24 hours were eligible to undergo a biovalidation test at that same visit, with the possibility of scheduling a follow-up biovalidation test for the following day with instructions not to smoke the previously reported other combustible substance or substances over the intervening 24-hour period. If a participant was eligible for biovalidation and biovalidation was not achieved, the reason was noted (eg, did not schedule or attend a biovalidation study visit, reported change in smoking status at the outset of visit, or the participant’s breath CO sample was ≥10 ppm).

Biovalidation was obtained through CO breath sampling. Participants in the intervention arm used their Pivot Breath Sensor for this test. Shortly before the visit, participants in the control arm were mailed a Pivot Breath Sensor that was limited to 10 breath samples. During the video call, participants held the breath sensor up to the screen immediately after completing the breath sample so that the study staff could see and record the CO ppm measurement on the sensor screen. A CO value of <10 ppm was considered consistent with abstinence [17,34,35].

### Outcomes and Measures

#### Baseline

The following variables were collected at baseline: demographic information (age, gender, race, ethnicity, household income, education, employment status, and smartphone type); smoking status; smoking history; Heaviness of Smoking Index [36]; success to quit (scale of 1-10); DTQ (scale of 1-10) [37,38]; and Smoking Abstinence Self-Efficacy Questionnaire (SASEQ), a 6-item survey describing emotional or social situations for which smokers indicate on a 5-point Likert scale (0-4) whether they will be able to refrain from smoking, with higher scores representing higher self-efficacy [39].

#### 52 Weeks

At the 52-week time point, the study outcomes focused on smoking behavior and self-efficacy. Assessments included the SASEQ; quit attempts; CPD; use of NRT; and smoking cessation via self-reported 7-day PPA, 30-day PPA, and continuous abstinence; biochemically confirmed abstinence; biochemically confirmed continuous abstinence; and self-reported abstinence from all tobacco products.

Self-efficacy was assessed using the SASEQ. Participants were considered to have made a quit attempt during the study if they answered ≥1 to the following question: “Since you began the study, how many times have you tried to quit smoking where you’ve gone at least 1 day without smoking a cigarette, even a single puff?” From this question, the mean quit attempts per participant were quantified as well. CPD were assessed through the mean percentage change and the proportion of participants who reduced their CPD by ≥50% compared with the baseline. NRT use included whether a participant ordered NRT (yes or no)—and, if so, the type of NRT they ordered—using participant-placed orders.

Participants were considered to have achieved self-reported 7-day (30-day) PPA if they answered “no” to the following question: “In the last 7 (30) days have you smoked any cigarettes, even a single puff?” Biochemically confirmed abstinence was defined as self-reporting 7-day abstinence and having a breath CO sample of <10 ppm at the 52-week biovalidation visit. Self-reported continuous abstinence was defined as self-reporting 7-day (or greater) PPA at 12 weeks, self-reporting 30-day PPA at 26 and 52 weeks, no more than 5 cigarettes between 12 and 26 weeks, and 0 cigarettes between 26 and 52 weeks. Biochemically confirmed continuous abstinence was defined as self-reported continuous abstinence, as detailed previously, with a breath CO sample of <10 ppm at each of the associated 12-, 26-, and 52-week biovalidation visits. Abstinence from all tobacco products was self-reported.

### Sample Size

As this was a pilot RCT and the first assessment of Pivot compared with usual care, the sample size was powered to show differences in engagement, specifically, the number of times participants opened their assigned app over the first 12 weeks of the study. The methodology to power the study for engagement and the associated outcomes have been previously reported. Pivot participants self-reported a mean of 157.9 (SD 210.6) total app openings versus 86.5 (SD 66.3) in the QuitGuide group (incidence rate ratio [IRR]=1.8, 95% CI 1.4-2.3; *P*<.001) through week 12 [17].

### Statistical Analyses

#### Comparisons

For results in which a change from baseline could be measured (CPD and SASEQ), each participant’s baseline data served as their control to then calculate a difference at 52 weeks, and a paired 2-tailed *t* test was used to test for a significant difference from 0. The outcomes were evaluated using regression analyses adjusted for the 4 randomization stratification covariates to detect differences between the treatment and control arms. Linear regression was used for numerical data to obtain a point estimate of the mean difference. For count outcomes, the IRR was estimated using Poisson regression when the variance-to-mean ratio was close to 1 or using negative binomial regressions when the variance-to-mean ratio was >1. For binary outcomes, the OR was estimated using logistic regression, and the RR was estimated using either log-link binomial regression or log-link Poisson regression with robust estimators [40]. For binary outcomes where there was a very high-frequency response (eg, ≥95%), only the RR was presented. For multicategory outcomes of ≥3, multinomial logistic regression was used to test for proportion differences between the study arms.

In the assessment of quit rates (self-reported and biovalidated PPA, self-reported and biovalidated continuous abstinence, and self-reported abstinence from all tobacco products), 2 sets of analyses were performed. In the ITT analysis, individuals who did not respond to the PPA questions were assumed to be smoking. A study responder analysis was also performed, which only included individuals who completed the 52-week questionnaire. For the outcomes of quit attempts and the proportion of participants who reduced CPD by ≥50%, a study responder analysis was performed.

#### Predictors

We also performed exploratory post hoc analyses that may help inform the design of future studies. We used logistic regression to examine the associations between baseline characteristics and smoking behavior outcomes at 52 weeks. Each independent baseline variable was evaluated as a predictor in either 1 or 2 types of models using the ITT and responder data sets. The first model evaluated the interaction of the independent baseline variable with the randomized cohort. The interaction was tested for significance compared with a model without the interaction using the joint test. If the interaction was significant, the results were reported. If not, then the second model without the interaction was applied. In this model, the independent baseline variable was adjusted for the randomization cohort, and a type-3 test was used for detection of statistical significance. One *P* value is presented, either from the joint test if significant or from the type-3 test. For statistically significant models with baseline characteristics with ≥3 categories, additional permutations of the reference were completed to appropriately characterize the difference.

This evaluation was completed for the binary outcomes of self-reported 7-day and 30-day PPA, biovalidated 7-day PPA, self-reported continuous abstinence, and biovalidated continuous abstinence. In contrast to the analysis of outcomes, these models were not adjusted for the 4 randomization variables. Certain categorical baseline variables were collapsed either because of insufficient tallies for model convergence or to simplify the model evaluation. These baseline variables included ethnicity, education, income, health, use of noncigarette tobacco products, past quit methods used, and first cigarette smoked after waking. In addition, some of the interaction models produced quasi-complete separation, and the data were modeled again with logistic regression using a Firth bias correction [41]. Analyses were conducted using SAS (version 9.4; SAS Institute). Statistical significance was set at *P*<.05.

### Data Collection

Data collection was performed using web-based questionnaires at baseline and at the 52-week follow-up. Study data were imported directly into a secure database (PostgreSQL; PostgreSQL Global Development Group).

Participants were compensated with US $50 for completing the 52-week web-based questionnaire and US $50 if they were eligible for and completed the associated biovalidation visit. Taking into consideration the 15 participant questionnaires conducted over the 52-week study period (with compensation of US $10-$50 per questionnaire) and the possibility of 2 previous biovalidation visits with compensation of US $50 each, participants could earn up to US $465 in total over the course of the 52-week study. Compensation was in the form of Visa or Mastercard gift cards that were emailed or mailed to their provided address approximately 2 to 3 weeks after completing the associated questionnaire or questionnaires or biovalidation visits. Remuneration was not tied to quitting smoking or use of one’s study program.

### Ethics Approval

The study was reviewed and approved by Solutions Institutional Review Board, LLC (protocol 2021/04/38) and registered with ClinicalTrials.gov (NCT04955639).

## Results

### Enrollment and Questionnaire Completion

From June 2021 to October 2021, a total of 3042 web-based screening forms were received; 533 (17.52%) met the screening eligibility criteria and responded to an initial outbound phone call from the study staff. Of these 533 individuals, 188 (35.3%) were randomized and completed enrollment (n=94, 50% in each arm), comprising the ITT sample (188/3042, 6.18%).

Of the 3042 potential participants who completed the web-based screening form, 1085 (35.67%) were ineligible.Among these 1085 individuals, the reasons for ineligibility were readiness to quit (n=619, 57.1% not ready to quit within the next 30 days), quota filled for employment category (n=115, 10.6%), incompatible phone (n=72, 6.6%), form completed by someone other than the potential participant (n=44, 4.1%), currently smoking <7 days per week (n=44, 4.1%), currently smoking <5 CPD (n=38, 3.5%), being aged <21 years (n=5, 0.5%), and having ≥2 disqualifications (n=148, 13.6%).

**Figure 1:**
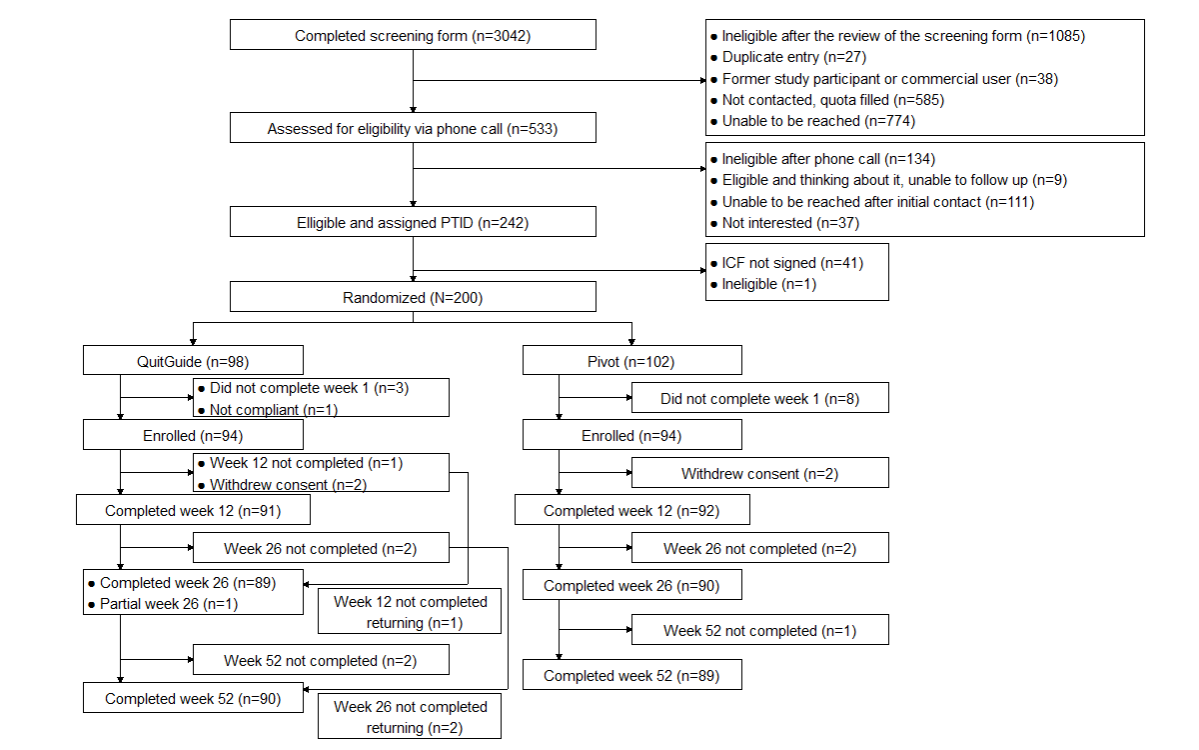
Study participant CONSORT (Consolidated Standards of Reporting Trials) flow diagram. ICF: informed consent form; PTID: participant identification number.

In each arm, 2% (2/94) of the participants withdrew consent within the first 3 weeks of the study. The 52-week study questionnaire was completed by 95.2% (179/188) of the participants, specifically by 95% (89/94) in the Pivot arm and 96% (90/94) in the QuitGuide arm; these comprise the study responder samples. Study enrollment and attrition are depicted in the CONSORT (Consolidated Standards of Reporting Trials) flow diagram (Figure 1).

### Baseline Characteristics

The study sample had a mean age of 46.4 (SD 9.2) years, comprised 55.3% (104/188) women, was predominantly White (128/188, 68.1%), smoked a mean of 17.6 (SD 9.0) CPD at baseline, and had been smoking for a mean of 26.8 (SD 10.3) years. The mean Heaviness of Smoking Index was 3.2 (SD 1.2). Participants represented 42 of the 50 states in the United States, along with the District of Columbia. The following states were not represented: Alaska, Delaware, Maine, Montana, North Dakota, New Hampshire, Vermont, and Wyoming. On average, participants had made 2.0 (SD 3.6) quit attempts over the 12 months before study entry. Baseline demographic characteristics and smoking behavior were balanced between the treatment groups at baseline. Participant baseline data are presented in Table 1.

**Table 1:** Participant baseline data (N=188).

Characteristic				All		Pivot (n=94)		QuitGuide (n=94)		*P* value
**Demographics**										
	Age (years), mean (SD)		46.4 (9.2)		46.6 (10.1)		46.1 (8.2)		.70	
	Gender (women), n (%)		104 (55.3)		50 (53.2)		54 (57.4)		.56	
	**Ethnicity and race, n (%)**									.52
		American Indian or Alaska Native	1 (0.5)		1 (1.1)		0 (0)			
		Asian	1 (0.5)		0 (0)		1 (1.1)			
		Black	36 (19.1)		15 (16)		21 (22.3)			
		Hispanic, Latino, or Spanish origin	13 (6.9)		8 (8.5)		5 (5.3)			
		Native Hawaiian	2 (1.1)		0 (0)		2 (2.1)			
		White	128 (68.1)		66 (70.2)		62 (66)			
		Some other race	3 (1.6)		2 (2.1)		1 (1.1)			
		Prefer not to answer	4 (2.1)		2 (2.1)		2 (2.1)			
	**Education, n (%)**									.64
		Less than eighth grade	1 (0.5)		0 (0)		1 (1.1)			
		Some high school	2 (1.1)		1 (1.1)		1 (1.1)			
		High school or GED^a^	27 (14.4)		15 (16)		12 (12.8)			
		Some college	80 (42.6)		35 (37.2)		45 (47.9)			
		Associate’s (2-year) degree	28 (14.9)		13 (13.8)		15 (16)			
		Bachelor’s (4-year) degree	31 (16.5)		18 (19.1)		13 (13.8)			
		Master’s degree	15 (8)		10 (10.6)		5 (5.3)			
		Professional or doctorate degree	4 (2.1)		2 (2.1)		2 (2.1)			
	**Income (US $), n (%)**									.34
		<25,000	32 (17)		14 (14.9)		18 (19.1)			
		25,000-34,999	26 (13.8)		14 (14.9)		12 (12.8)			
		35,000-49,999	42 (22.3)		19 (20.2)		23 (24.5)			
		50,000-74,999	32 (17)		13 (13.8)		19 (20.2)			
		75,000-99,999	23 (12.2)		12 (12.8)		11 (11.7)			
		100,000-149,999	15 (8)		8 (8.5)		7 (7.4)			
		≥150,000	10 (5.3)		8 (8.5)		2 (2.1)			
		Prefer not to answer	8 (4.3)		6 (6.4)		2 (2.1)			
	**Employment, n (%)**									.83
		Yes, ≥20 h/wk	117 (62.2)		59 (62.8)		58 (61.7)			
		Yes, <20 h/wk	37 (19.7)		17 (18.1)		20 (21.3)			
		No	34 (18.1)		18 (19.1)		16 (17)			
	**Self-reported health, n (%)**									.35
		Excellent	5 (2.7)		4 (4.3)		1 (1.1)			
		Very good	57 (30.3)		24 (25.5)		33 (35.1)			
		Good	99 (52.7)		51 (54.3)		48 (51.1)			
		Fair	26 (13.8)		14 (14.9)		12 (12.8)			
		Poor	1 (0.5)		1 (1.1)		0 (0)			
	**Smartphone, n (%)**									.30
		iPhone	113 (60.1)		60 (63.8)		53 (56.4)			
		Android	75 (39.9)		34 (36.2)		41 (43.6)			
**Smoking and quitting behavior**										
	Cigarettes smoked per day, mean (SD)		17.6 (9.0)		18.0 (9.6)		17.2 (8.5)		.55	
	Years smoking, mean (SD)		26.8 (10.3)		27.7 (10.4)		25.8 (10.1)		.21	
	**First cigarette smoked after waking, n (%)**									.52
		Within 5 min	67 (35.6)		30 (31.9)		37 (39.4)			
		6 to 30 min	92 (48.9)		47 (50)		45 (47.9)			
		31 to 60 min	22 (11.7)		12 (12.8)		10 (10.6)			
		After 60 min	7 (3.7)		5 (5.3)		2 (2.1)			
	**Tobacco products used, n (%)**									.42
		Cigarettes only	162 (86.2)		79 (84)		83 (88.3)			
		Cigarettes+e-cigarettes or vaping	15 (8)		10 (10.6)		5 (5.3)			
		Cigarettes+cigars	3 (1.6)		1 (1.1)		2 (2.1)			
		Cigarettes+e-cigarettes or vaping+cigars	2 (1.1)		1 (1.1)		1 (1.1)			
		Cigarettes+chew or snuff	2 (1.1)		2 (2.1)		0 (0)			
		Cigarettes+e-cigarettes, vaping+chew, or snuff	1 (0.5)		0 (0)		1 (1.1)			
		Cigarettes+e-cigarettes or vaping+pipe	1 (0.5)		0 (0)		1 (1.1)			
		Cigarettes+hookah+cigars	1 (0.5)		0 (0)		1 (1.1)			
		Cigarettes+hookah	1 (0.5)		1 (1.1)		0 (0)			
	HSI^b^, mean (SD)		3.2 (1.2)		3.2 (1.3)		3.2 (1.2)		.72	
	Quit attempts in the past 12 months, mean (SD)		2.0 (3.6)		1.9 (3.4)		2.2 (3.8)		.63	
	**Methods used in past quit attempts^c^, n (%)**									—^d^
		Cold turkey	140 (74.5)		67 (71.3)		73 (77.7)			
		NRT^e^	92 (48.9)		53 (56.4)		39 (41.5)			
		e-Cigarettes or vaping	65 (34.6)		33 (35.1)		32 (34)			
		Varenicline or Chantix	50 (26.6)		23 (24.5)		27 (28.7)			
		Bupropion, Zyban, or Wellbutrin	33 (17.6)		26 (27.7)		7 (7.4)			
		None	16 (8.5)		5 (5.3)		11 (11.7)			
		Hypnotherapy	11 (5.9)		7 (7.4)		4 (4.3)			
		Classes for quitting smoking	10 (5.3)		7 (7.4)		3 (3.2)			
		Acupuncture	10 (5.3)		7 (7.4)		3 (3.2)			
		Smartphone app	9 (4.8)		6 (6.4)		3 (3.2)			
		Counseling	4 (2.1)		2 (2.1)		2 (2.1)			
		Other	4 (2.1)		1 (1.1)		3 (3.2)			
	**Attitudes toward quitting smoking, mean (SD)**									—
		DTQ^f^	3.5 (2.5)		3.5 (2.3)		3.6 (2.6)		.72	
		STQ^g^	4.5 (2.4)		4.6 (2.4)		4.3 (2.3)		.33	
		SASEQ^h^	11.7 (4.8)		11.8 (4.7)		11.5 (4.9)		.69	

^a^GED: general educational development.

^b^HSI: Heaviness of Smoking Index; low (0-1), medium (2-4), and high (5-6).

^c^Participants were asked to select all that applied.

^d^Not available.

^e^NRT: nicotine replacement therapy.

^f^DTQ: difficulty to stay quit; if you were to quit smoking right now, how difficult do you think it would be to stay smoke free? (1=really hard to stay quit; 10=really easy to stay quit).

^g^STQ: success to quit; if you were to quit smoking right now, how successful would you be? (1=not at all successful; 10=completely successful).

^h^SASEQ: Smoking Abstinence Self-Efficacy Questionnaire (score of 1-24).

### Self-Efficacy

The SASEQ increased in both groups from baseline to week 52 (Table 2). The increase in the Pivot group of 3.2 (SD 7.8) points was significant (*P*<.001), whereas the increase in the QuitGuide group of 1.0 (SD 8.5) points was not (*P*=.26).

**Table 2:** Changes in the Smoking Abstinence Self-Efficacy Questionnaire (SASEQ) from baseline to 52 weeks (N=188).

SASEQ^a^	All			Pivot			QuitGuide			*P* value^b^		Point estimate^c^ (95% CI)		*P* value^c^
	Participants, n (%)	Values, mean (SD)^d^	Participants, n (%)		Values, mean (SD)^e^	Participants, n (%)		Values, mean (SD)^f^						
Baseline	188 (100)	11.7 (4.8)	94 (50)		11.8 (4.7)	94 (50)		11.5 (4.9)	.69		N/A^g^		N/A	
52 weeks	179 (95.2)	13.7 (8.1)	89 (47.3)		14.9 (7.9)	90 (47.9)		12.6 (8.1)	N/A		2.3 (0.01 to 4.6)		.049	
Change	N/A	2.1 (8.2)	N/A		3.2 (7.8)	N/A		1.0 (8.5)	N/A		2.2 (−0.1 to 4.5)		.06	

^a^SASEQ score of 1-24.

^b^2-tailed *t* test between Pivot and QuitGuide.

^c^Point estimate and corresponding *P* value obtained from linear regression adjusted with randomization covariates: daily smoking frequency (≤14 vs ≥15 cigarettes per day), employment status (full-time or part-time employment vs not employed), race and ethnicity (minority race and ethnicity vs non-Hispanic White), and expected difficulty staying quit (scale of 1-10; self-reported score of ≤5 vs ≥6).

^d^*P*<.001 (paired *t* test change, ie, difference from baseline to week 52).

^e^*P*<.001 (paired *t* test change, ie, difference from baseline to week 52).

^f^*P*=.26 (paired *t* test change, ie, difference from baseline to week 52).

^g^N/A: not applicable.

### Smoking Behavior

#### Quit Attempts

Using an ITT analysis, at 52 weeks, 96.8% (182/188) of participants reported having made at least one quit attempt since the study started, with comparable proportions in each study group (90/94, 96% for Pivot and 92/94, 98% for QuitGuide; OR 0.5, 95% CI 0.1-2.8, *P*=.44; RR Poisson=1.0, 95% CI 0.9-1.0, *P*=.42). On average, QuitGuide participants reported more quit attempts (mean 7.0, SD 6.3 for Pivot vs 9.5, SD 7.5 for QuitGuide; IRR negative binomial=0.7, 95% CI 0.6-0.9; *P*=.01).

#### Change in CPD

Among participants who responded at 52 weeks (179/188, 95.2%), CPD were reduced by 63.4% (SD 39.3%) from baseline. Within each group, the reduction in CPD from baseline to week 52 was significant (*P*<.001 for both). The CPD reduction was similar between the 2 groups (mean −63.3%, SD 42% for Pivot vs −63.4%, SD 36.7% for QuitGuide; point estimate=0.5, 95% CI −10.8 to 11.9; *P*=.93).

Among the subset of participants who did not report 7-day (or greater) PPA at 52 weeks (111/188, 59%), CPD were reduced by 40.9% (SD 34.1%) from baseline. Within each group, the reduction in CPD from baseline to week 52 was significant (*P*<.001 for both). The reduction in CPD was greater in the QuitGuide group (mean −34.6%, SD 35.5% vs −46.1%, SD 32.3% for QuitGuide; point estimate=13.1, 95% CI 0.6-25.7; *P*=.04).

Among the participants who responded at 52 weeks (179/188, 95.2%), the proportion who reduced CPD by ≥50% was similar between the 2 groups (58/89, 65% for Pivot vs 63/90, 70% for QuitGuide; OR 0.8, 95% CI 0.4-1.5, *P*=.44; RR Poisson=0.9, 95% CI 0.8-1.1, *P*=.44).

Focusing on the subset of participants who did not report 7-day (or greater) PPA at 52 weeks (111/188, 59%), the proportion of those who reduced CPD by ≥50% was 38% (19/50) for Pivot versus 56% (34/61) for QuitGuide (OR 0.4, 95% CI 0.2-0.9, *P*=.04; RR=0.7, 95% CI 0.4-1.0, *P*=.07).

#### Cessation Rates

Cessation rates are detailed in Table 3, which includes previously published rates from 26 weeks for context [17]. At 52 weeks, differences between the 2 study groups in self-reported 7- and 30-day PPA rates and abstinence from all tobacco products were not statistically significant (all *P*>.05). In contrast, self-reported continuous abstinence, biochemically confirmed abstinence, and biochemically confirmed continuous abstinence rates were significantly higher in the Pivot group (all *P*<.05).

Self-reported continuous abstinence (ITT) was achieved in 20% (19/94) of the Pivot participants versus 9% (8/94) of the QuitGuide participants (OR 2.8, 95% CI 1.1-6.9, *P*=.03; RR=2.4, 95% CI 1.1-5.1, *P*=.03). Biochemically confirmed abstinence (ITT) was achieved in 31% (29/94) of the Pivot participants versus 18% (17/94) of the QuitGuide participants (OR 2.1, 95% CI 1.0-4.3, *P*=.04; RR=1.9, 95% CI 1.1-3.1, *P*=.02). Biochemically confirmed continuous abstinence (ITT) was achieved in 19% (18/94) of the Pivot participants versus 9% (8/94) of the QuitGuide participants (OR 2.6, 95% CI 1.1-6.5, *P*=.04; RR=2.2, 95% CI 1.0-4.9, *P*=.046).

Notably, the participation rate in the 52-week biovalidation visit was 75% (51/68) overall—79% (31/39) in the Pivot group and 69% (20/29) in the QuitGuide group (OR 1.5, 95% CI 0.5-4.8, *P*=.51; RR=Poisson 1.1, 95% CI 0.8-1.5, *P*=.63). The 5 study participants (n=2, 40% in the Pivot group and n=3, 60% in the QuitGuide group) who completed a biovalidation visit but did not achieve biovalidated abstinence all reported a change in smoking status at the outset of the visit and, therefore, did not provide a breath sample during the visit. At the 52-week visit, no study participants had a breath sample value that was discordant with their self-reported abstinence.

**Table 3:** Smoking cessation rates at 26 and 52 weeks (N=188).

Outcome			Overall, n (%)		Pivot (n=94), n (%)		QuitGuide (n=94), n (%)		Adjusted OR^a^ (95% CI)		OR *P* value		RR^b^ (95% CI)		RR *P* value
**7-day PPA^c^**															
	52-week ITT^d^	68 (36.2)		39 (41.5)		29 (30.9)		1.6 (0.9-3.0)		.12		1.3 (0.9-2.0)		.14	
	26-week ITT	59 (31.4)		34 (36.2)		25 (26.6)		1.7 (0.9-3.2)		.12		1.4 (0.9-2.1)^e^		.12	
	52-week responder analysis^f^	68 (38)		39 (43.8)		29 (32.2)		1.6 (0.9-3.0)		.11		1.3 (0.9-2.0)		.15	
	26-week responder analysis^g^	59 (32.8)		34 (37.8)		25 (27.8)		1.7 (0.9-3.2)		.13		1.5 (1.0-2.3)		.06	
**30-day PPA**															
	52-week ITT	62 (33)		34 (36.2)		28 (29.8)		1.4 (0.7-2.5)		.33		1.2 (0.8-1.9)		.34	
	26-week ITT	51 (27.1)		30 (31.9)		21 (22.3)		1.7 (0.9-3.4)		.12		1.4 (0.9-2.2)		.18	
	52-week responder analysis	62 (34.6)		34 (38.2)		28 (31.1)		1.4 (0.7-2.6)		.32		1.2 (0.8-1.9)		.34	
	26-week responder analysis	51 (28.3)		30 (33.3)		21 (23.3)		1.7 (0.9-3.4)		.13		1.4 (0.9-2.22)		.19	
**Biovalidated abstinence**															
	52-week ITT	46 (24.5)		29 (30.9)		17 (18.1)		2.1 (1.0-4.3)		.04		1.9 (1.1-3.1)		.02	
	26-week ITT	40 (21.3)		26 (27.7)		14 (14.9)		2.3 (1.1-4.8)		.03		1.9 (1.1-3.5)		.02	
	52-week responder analysis	46 (25.7)		29 (32.6)		17 (18.9)		2.1 (1.1-4.3)		.04		1.9 (1.1-3.1)		.02	
	26-week responder analysis	40 (22.2)		26 (28.9)		14 (15.6)		2.3 (1.1-4.8)		.03		1.9 (1.1-3.4)		.02	
**Self-reported continuous abstinence**															
	52-week ITT	27 (14.4)		19 (20.2)		8 (8.5)		2.8 (1.1-6.9)		.03		2.4 (1.1-5.1)^e^		.03	
	26-week ITT	39 (20.7)		24 (25.5)		15 (16)		1.9 (0.9-3.8)		.10		1.6 (0.9-2.8)		.11	
	52-week responder analysis	27 (15.1)		19 (21.3)		8 (8.9)		2.8 (1.1-6.8)		.03		2.3 (1.1-5.0)		.03	
	26-week responder analysis	39 (21.7)		24 (26.7)		15 (16.7)		1.8 (0.9-3.9)		.11		1.6 (0.9-2.8)		.12	
**Biovalidated continuous abstinence**															
	52-week ITT	26 (13.8)		18 (19.1)		8 (8.5)		2.6 (1.1-6.5)		.04		2.2 (1.0-4.9)^e^		.046	
	26-week ITT	29 (15.4)		20 (21.3)		9 (9.6)		2.7 (1.1-6.4)		.03		2.2 (1.1-4.6)		.03	
	52-week responder analysis	26 (14.5)		18 (20.2)		8 (8.9)		2.6 (1.04-6.4)		.04		2.2 (1.0-4.8)		.047	
	26-week responder analysis	29 (16.1)		20 (22.2)		9 (10)		2.7 (1.1-6.3)		.03		2.3 (1.1-4.7)		.02	
**Self-reported abstinence from all tobacco products**															
	52-week ITT	60 (31.9)		33 (35.1)		27 (28.7)		1.4 (0.7-2.6)		.33		1.2 (0.8-1.8)		.41	
	26-week ITT	55 (29.3)		32 (34)		23 (24.5)		1.6 (0.9-3.1)		.13		1.5 (1.0-2.3)		.06	
	52-week responder analysis	60 (33.5)		33 (37.1)		27 (30)		1.4 (0.7-2.6)		.33		1.2 (0.8-1.8)		.44	
	26-week responder analysis	55 (30.6)		32 (35.6)		23 (25.6)		1.6 (0.8-3.1)		.16		1.5 (1.0-2.2)		.08	

^a^OR: odds ratio.

^b^RR: adjusted relative risk using log-link binomial regression unless otherwise noted (eg, it does not converge and log-link Poisson regression is used).

^c^PPA: point-prevalence abstinence.

^d^ITT: intention-to-treat analysis at 52 and 26 weeks; N=188 (n=94, 50% in the Pivot group and n=94, 50% in the QuitGuide group).

^e^Log-link Poisson regression used.

^f^Responder analysis at 52 weeks; n=179 (n=89, 49.7% in the Pivot group and n=90, 50.3% in the QuitGuide group).

^g^Responder analysis at 26 weeks; n=180 (n=90, 50% in the Pivot group and n=90, 50% in the QuitGuide group).

#### Use of NRT

At 52 weeks, 99% (93/94) of the Pivot participants had ordered NRT compared with 85% (80/94) of the QuitGuide participants (RR Poisson=1.2, 95% CI 1.1-1.3; *P*<.001). The average number of NRT orders placed per participant was 3.3 (SD 2.0) in the Pivot group and 1.8 (SD 1.6) in the QuitGuide group (IRR=1.8, 95% CI 1.5-2.2; *P*<.001). Combination therapy (patch+gum or patch+lozenge) was the most common regimen among participants (Table 4).

**Table 4:** Nicotine replacement therapy (NRT) orders placed by participants through 52 weeks (*P*<.001; N=188)^a^.

NRT order type	All, n (%)	Pivot (n=94), n (%)	QuitGuide (n=94), n (%)
≥1 NRT single therapy^b^ order	31 (16.5)	22 (23.4)	9 (9.6)
≥1 NRT combination therapy^c^ order	99 (52.7)	42 (44.7)	57 (60.6)
≥1 NRT single therapy+≥1 NRT combination therapy order	43 (22.9)	29 (30.9)	14 (14.9)
None	15 (8)	1 (1.1)	14 (14.9)

^a^Multinomial logistic regression adjusted for randomization covariates.

^b^Single therapy: nicotine patch alone, nicotine gum alone, or nicotine lozenge alone.

^c^Combination therapy: nicotine patch+nicotine gum or nicotine patch+nicotine lozenge.

### Participant Feedback

Among participants who reported 7-day PPA at 52 weeks (68/188, 36.2%), most reported that their study program helped them quit smoking (true or false response; 35/39, 90% in the Pivot group vs 26/29, 90% in the QuitGuide group; RR Poisson=1.0, 95% CI 0.9-1.2; *P*=.73).

### Predictors

We performed exploratory post hoc analyses using logistic regression to explore the qualitative associations between baseline characteristics and smoking behavior outcomes (Multimedia Appendix 1). We identified 3 variables with statistically significant correlation: age, education, and DTQ. None of these variables were considered predictive across all 5 smoking cessation outcomes.

Older age at baseline was associated with a decrease in self-reported 7- and 30-day PPA after adjusting for the randomization cohort. In addition, age had an interaction with cohort such that, in Pivot participants, older age was associated with achieving self-reported and biochemically confirmed continuous abstinence. In contrast, older age in QuitGuide participants was associated with a decreased likelihood of self-reported and biochemically confirmed continuous abstinence.

Regarding education, those who did not have any college experience were more likely to achieve biochemically confirmed abstinence compared with those with some college, a 2-year degree, or ≥4 years of college.

Finally, contrasting correlations were observed for DTQ in each cohort. In Pivot participants, a higher DTQ at entry was associated with a decreased likelihood of achieving self-reported 30-day PPA. In QuitGuide participants, a higher DTQ at entry was associated with an increased likelihood of self-reported 30-day PPA.

## Discussion

### Principal Findings

In this paper, we report the results at 52 weeks of this pilot RCT comparing the Pivot and QuitGuide mobile smoking cessation programs among 188 adult cigarette smokers in the United States. Pivot had higher smoking cessation rates for self-reported continuous abstinence (19/94, 20% for Pivot vs 8/94, 9% for QuitGuide; *P*=.03), biochemically confirmed abstinence (29/94, 31% for Pivot vs 17/94, 18% for QuitGuide; *P*=.04), and biochemically confirmed continuous abstinence (18/94, 19% for Pivot vs 8/94, 9% for QuitGuide; *P*=.046). Pivot participants also had a significant increase in self-efficacy via the SASEQ, whereas QuitGuide participants did not. In addition, more Pivot participants (93/94, 99% in the Pivot group vs 80/94, 85% in the QuitGuide group; *P*<.001) placed NRT orders (Pivot mean 3.3, SD 2.0 vs QuitGuide mean 1.8, SD 1.6; *P*<.001).

On average, QuitGuide participants made more quit attempts (7.0, SD 6.3 for Pivot vs 9.5, SD 7.5 for QuitGuide; *P*=.01). Among the subset of participants who did not report 7-day (or greater) PPA at 52 weeks, QuitGuide participants had a greater reduction in CPD (−34.6% for Pivot vs −46.1% for QuitGuide; *P*=.04). Among those who reported 7-day PPA at 52 weeks, most in each arm (35/39, 90% in the Pivot group and 26/29, 90% in the QuitGuide group) indicated that their study program helped them quit smoking.

### Smoking Cessation Rates: Comparison With Prior Work

The availability of comparative long-term data from RCTs assessing mobile smoking cessation programs is limited, as shown in Table 5. At 1 year, 7-day PPA rates were similar between this study and the RCT by Webb et al [16] assessing Quit Genius (39/94, 42% in the Pivot group and 35% in the Quit Genius group) and slightly higher than the 29% reported by Bricker et al [14] for iCanQuit. A similar pattern was reported for durable abstinence rates at 1 year (20%-22% for this study and the RCT by Webb et al [16] and 10% for the RCT by Bricker et al [14]). Notably, this study and the RCT by Webb et al [16] provided participants with NRT; the RCT by Bricker et al [14] did not. This may have contributed to the differences in cessation rates.

**Table 5:** Comparative 1-year smoking cessation outcomes from randomized controlled trials assessing mobile smoking cessation programs.

Study	Outcome													
	7-day PPA^a^, n/N (%)			Statistical analysis		30-day PPA, n/N (%)			Statistical analysis		Durable abstinence, n/N (%)			Statistical analysis
	Intervention	Control			Intervention		Control			Intervention		Control		
Bricker et al^b^ [14]	356/1214 (29.3)	302/1201 (25.1)	OR^c^ 1.26, 95% CI 1.05-1.52; *P*=.01		293/1214 (24.1)		225/1201 (18.7)	OR 1.40, 95% CI 1.14-1.71; *P*=.001		116/1214^d^ (9.6)		65/1201 (5.4)	OR 1.84, 95% CI 1.34-2.53; *P*<.001	
Webb et al^e^ [16]	92/265 (34.7)	78/265 (29.4)	IRR^f^ 1.20, 95% CI 0.94-1.54; *P*=.19		Not assessed		Not assessed	N/A^g^		58/265^h^ (21.9)		30/265 (11.3)	RR^i^ 1.93, 95% CI 1.29-2.90; *P*=.002	
Marler et al^j^ [17]	39/94 (41.5)	29/94 (30.9)	OR 1.6, 95% CI 0.9-3.0; *P*=.12		34/94 (36.2)		28/94 (30)	OR 1.4, 95% CI 0.7-2.5; *P*=.33		19/94^k^ (20.2)		8/94 (8.5)	OR 2.8, 95% CI 1.1-6.9; *P*=.03	

^a^PPA: point-prevalence abstinence. Participants were considered to have achieved self-reported 7-day (30-day) PPA if they answered “no” to the following question: “In the last 7 (30) days have you smoked any cigarettes, even a single puff?”

^b^Intervention: iCanQuit; control: QuitGuide.

^c^OR: odds ratio.

^d^Prolonged abstinence defined as the time since the date of the last cigarette. The dates of the last cigarette that occurred between 0 and 90 days after randomization were categorized as prolonged abstinence. Dates that occurred ≥91 days after randomization were categorized as not prolonged abstinence.

^e^Intervention: Quit Genius; control: very brief advice.

^f^IRR: incidence rate ratio.

^g^N/A: not applicable.

^h^Sustained abstinence was defined as smoking no more than 5 cigarettes from the quit date to the 52-week follow-up.

^i^RR: relative risk.

^j^Intervention: Pivot; control: QuitGuide.

^k^Continuous abstinence was defined as self-reporting 7-day (or greater) PPA at 12 weeks, self-reporting 30-day PPA at 26 and 52 weeks, no more than 5 cigarettes between 12 and 26 weeks, and 0 cigarettes between 26 and 52 weeks.

### Differences in Other Smoking-Related Behaviors and Outcomes Between Pivot and QuitGuide

There were notable differences in other smoking-related outcomes and behaviors between the 2 study groups. Specifically, at 52 weeks, participants in the Pivot group reported a significant increase in self-efficacy via the SASEQ, whereas QuitGuide participants did not. Moreover, more Pivot participants ordered NRT over the course of the study, with higher mean NRT orders per person. Both higher self-efficacy and NRT use are associated with an increased likelihood of cessation [26,42-46], and these factors may have contributed to the higher cessation rates in the Pivot group.

QuitGuide participants made, on average, more quit attempts and achieved a greater reduction in CPD among individuals who did not report abstinence. The literature provides a mixed picture of quit attempts, acknowledging that it can take an average of 30 quit attempts before successfully quitting for 1 year or longer [47]. By this logic, more quit attempts bring one closer to quitting, and setting realistic expectations accordingly may be helpful. However, there have also been reports of psychological distress, anxiety, and frustration that come with failed quit attempts [48-54]. Together, these data suggest that both the quantity and quality of quit attempts are important factors in successful cessation. A reduction in CPD has been reported as a meaningful behavior change that increases the probability of future cessation [55]. As the study participants continue surveillance, it remains to be seen whether this outcome in the QuitGuide group will translate to improved quit rates in the future.

### Predictors

Exploratory post hoc analyses using logistic regression identified age, education, and DTQ as baseline variables associated with smoking cessation outcomes. Considering the exploratory nature of these analyses, the relatively small number of participants in the different baseline categories, and that none of these baseline variables had statistically significant correlations for all 5 smoking cessation outcomes, caution should be used in the interpretation of these data. Accordingly, we have limited our interpretations to a qualitative assessment. However, a review of the literature also reveals previous identification of these variables as associated with smoking cessation.

Several studies have reported that older age is associated with smoking cessation [56-58], which was true in the Pivot group for self-reported and biochemically confirmed continuous abstinence. Interestingly, in the QuitGuide group, older age was associated with a lower likelihood of self-reported and biochemically confirmed continuous abstinence. This finding has been reported elsewhere; however, the age effect disappeared when controlling for heaviness of smoking [59].

The finding that a lower educational level (no college experience) was associated with a higher likelihood of biochemically confirmed abstinence has also been reported elsewhere [60,61]. However, reports on the association between higher educational levels and successful cessation are more common [19,57,58,62-64].

Finally, a higher DTQ at study entry was associated with a lower likelihood of self-reported 30-day PPA in the Pivot group and a higher likelihood in the QuitGuide group. Approaching the DTQ metric used in this study as a general proxy for self-efficacy, the literature predominantly supports the association between higher self-efficacy and a greater likelihood of cessation and between lower self-efficacy and a lower likelihood of cessation and a greater likelihood of relapse [19,42-46].

Overall, the data related to predictors of cessation are nuanced and likely heavily influenced by the presence, type, and use of a cessation program and by the cessation program characteristics. For example, some populations may respond better to specific program tools or delivery mechanisms than others. The value of these analyses is that they highlight variables of interest for future study designs and assist in teasing out optimal pairings between populations and cessation approaches.

### Strengths and Limitations

This study has several strengths. First, nonproportional quota sampling achieved a diverse and balanced population. The smoking cessation programs compared in this study were also of the same modality, decreasing the likelihood of modality-related confounding. In addition, QuitGuide as a control program provides sufficient context for the study outcomes as a well-established and well-studied digital cessation program [14,15,17,33]. Another strength of this study is the inclusion of 3 separate biovalidation visits for those who reported at least 7-day abstinence at 12, 26, and 52 weeks, providing validation for self-reported claims of smoking abstinence. In addition, the 52-week follow-up provided additional insights into the effectiveness of each program for smoking cessation on a longer-term scale. Finally, the following measures of study participation remained robust for 52 weeks: retention (approximately 89/94, 95% in each arm), survey completion (≥92%; ≥87/94 for each survey), and biovalidation visit completion (151/186, 81.2% overall).

This study also has several limitations. First, as a pilot study, it was not powered for cessation outcomes. The self-reported PPA and abstinence from all tobacco products outcomes were not significantly different between the 2 study groups, whereas the biovalidated abstinence and self-reported continuous abstinence outcomes were. Whether a larger study powered for these outcomes would have resulted in significant differences in smoking abstinence outcomes is unknown.

Second, the Pivot program includes the following additional tools not included in the QuitGuide program: a CO breath sensor, SMS text messaging–based counseling with a tobacco cessation coach, and a moderated web-based community support forum. This study compares the 2 programs but cannot determine the specific effects of these additional tools. Accordingly, the appropriate focus of future assessments includes whether and to what extent these tools contribute to Pivot outcomes.

Third, we cannot rule out some influence on participant selection by the recruiting and enrollment process and how this might influence the generalizability of study outcomes to the population of smokers as a whole. We took steps to mitigate factors that might incentivize enrollment, such as the availability of free NRT and study compensation. Specifically, these study characteristics were not mentioned in the recruiting advertisements or web-based screening form or until later in the screening phone calls. Potential participants were deemed ineligible if they were already using NRT or other smoking cessation tools at study entry. Steps were also taken to minimize the possible influence of compensation, including not tying compensation to outcome or program use, delaying payments 2 to 3 weeks after the completion of compensated events, and keeping payment amounts conservative. Recruitment was conducted on social media throughout the United States, and ultimately, study participants represented 42 of the 50 states, along with the District of Columbia. We also used nonproportional quota sampling for several participant characteristics (age group, gender, race, and employment status), with potential participants called on a first-come, first-served basis accordingly. All nonproportional quota sampling goals were met. Nonetheless, it is always important to consider factors that may influence the generalizability of study outcomes to larger populations.

Fourth, after randomization, all researchers were unblinded to participant group allocation, which can result in unbalanced participant communication and data collection efforts. To mitigate this, the study design included scheduled, standardized, and scripted participant communications (written and verbal) reviewed by the institutional review board. High and comparable questionnaire and biovalidation visit completion rates (≥87/94, ≥92% for questionnaires and ≥69% for biovalidation visits at 12, 26, and 52 weeks in both study arms) reflect favorably on our attempt to minimize these possible effects.

Fifth, exhaled CO as a biovalidation test for smoking cessation is imperfect. The half-life of CO is, on average, 4 hours and is influenced by activity level (ie, shorter half-life when exercising and longer half-life when sleeping). Accordingly, smokers may be able to abstain from smoking for several hours before providing a breath sample and obtain a CO value consistent with “not smoking”; we cannot exclude this occurrence during the biovalidation visits. Moreover, secondhand smoke, use of other combustible substances such as marijuana, and environmental or occupational CO exposure can increase CO levels. That said, the limitations of other biovalidation methods made exhaled CO, which is noninvasive, less expensive, and easy for a lay user to perform, the preferred option. Specifically, although cotinine, a nicotine metabolite, has a longer half-life (≥8-30 hours) than CO and, therefore, requires longer abstinence periods (2-7 days) to reach “nonsmoking” levels, its collection from body fluids is more onerous and will yield positive results in individuals using NRT, which was problematic with our study design. Anabasine and anatabine are minor tobacco alkaloids that are specific for tobacco-derived products (eg, cigarettes, cigars, and smokeless tobacco). They are well suited for testing individuals who use NRT for tobacco use. However, these biomarkers require urine collection and chromatography–mass spectrometry measurement [65]. Altogether, when considering the remote nature of this study and the provision of NRT, we felt that exhaled CO, despite its imperfections, was the best option for biovalidation.

Finally, this study did not address the reported differential cessation outcomes in the context of cost-effectiveness. Although it is beyond the scope of this study, a cost-benefit analysis is an appropriate and logical next step. Resource use is an important consideration in the assessment of mobile tobacco cessation interventions. We have sought to establish foundational outcomes in Pivot; these inputs should be included in future cost-benefit assessments.

### Conclusions

In this pilot RCT comparing the Pivot and QuitGuide mobile smoking cessation programs, Pivot had higher abstinence rates at 52 weeks. The context of this outcome in a study powered for engagement underscores the effectiveness of the intervention. Moreover, the treatment effect was durable, with stable biovalidated continuous abstinence rates from 26 to 52 weeks. This study adds to the small but growing body of RCT-derived evidence focused on longer-term outcomes among adult smokers using mobile smoking cessation programs. The data are encouraging and consistent, pointing to an important role for these types of programs in the larger effort to curb nicotine dependence.

## Appendix

Multimedia Appendix 1 Intention-to-treat (N=188) logistic regression analyses of baseline predictors of 7-day point-prevalence abstinence (PPA), 30-day PPA, biovalidated PPA, continuous abstinence, and biovalidated continuous abstinence at 52 weeks among all study participants.

Multimedia Appendix 2 CONSORT-EHEALTH Checklist (V 1.6.1).

## Abbreviations

CO: carbon monoxide
CONSORT: Consolidated Standards of Reporting Trials
CPD: cigarettes per day
DTQ: difficulty to stay quit
FDA: Food and Drug Administration
HIPAA: Health Insurance Portability and Accountability Act
IRR: incidence rate ratio
ITT: intention to treat
NCI: National Cancer Institute
NRT: nicotine replacement therapy
OR: odds ratio
PPA: point-prevalence abstinence
ppm: parts per million
RCT: randomized controlled trial
RR: relative risk
SASEQ: Smoking Abstinence Self-Efficacy Questionnaire
USCPG: United States Clinical Practice Guideline

## Data Availability

Deidentified data will be shared upon reasonable request stating the purpose to the corresponding author, which is subject to approval by the sponsor.
