# Supplementary material for: Long-Term Outcomes of a Comprehensive Mobile Smoking Cessation Program With Nicotine Replacement Therapy in Adult Smokers: Pilot Randomized Controlled Trial
Source: JMIR Mhealth Uhealth. 2023 Sep 18;11:e48157. doi: 10.2196/48157 (PMC10546267; doi:10.2196/48157)
Supplement: Multimedia Appendix 1 [file mhealth_v11i1e48157_app1.docx]

Multimedia Appendix 1. ITT (N=188) Logistic regression analyses of baseline predictors of 7-day point prevalence abstinence (PPA), 30-day PPA, biovalidated PPA, continuous abstinence and biovalidated continuous abstinence at 52 weeks among all study participants.

|  | **All (N)** | **Pivot (n)^a^** | **Quit-Guide (n)^a^** | **7-day PPA^b^** | ***P^b^*** | **30-day PPA^b^** | ***P^b^*** | **Biovalidated continuous abstinence^b^** | ***P^b^*** | **Self-reported continuous abstinence^b^** | ***P^b^*** | **Biovalidated abstinence^b^** | ***P^b^*** |
| --- | --- | --- | --- | --- | --- | --- | --- | --- | --- | --- | --- | --- | --- |
| Cohort |  |  |  | 1.6  (0.9-2.9) | 0.13 | 1.4 (0.7-2.5) | 0.35 | 2.1 (1.0-4.1) | 0.04 | 2.7 (1.1-6.5) | 0.03 | 2.5 (1.0 - 6.1) | 0.04 |
| Age (years) | 188 | 94 | 94 | 1 (0.9-1) | 0.04 | 1 (0.9-1) | 0.04 | 1 (0.9-1) | 0.15 | PV 1 (0.96-1.1) QG 0.9 (0.8-0.99) | 0.03 | PV 1 (0.96-1.1) QG 0.9 (0.8-0.99) | 0.03 |
| Gender |  |  |  |  |  |  |  |  |  |  |  |  |  |
| Male | 84 | 44 | 40 | 1 [Ref]^c^ | 0.67 | 1 [Ref] | 0.50 | 1 [Ref] | 0.68 | 1 [Ref] | 0.90 | 1 [Ref] | 0.94 |
| Female | 104 | 50 | 54 | 0.9 (0.5-1.6) |  | 0.8 (0.4-1.5) |  | 0.9 (0.4-1.7) |  | 1.1 (0.5-2.4) |  | 1 (0.4-2.2) |  |
| Ethnicity |  |  |  |  |  |  |  |  |  |  |  |  |  |
| White | 128 | 66 | 62 | 1 [Ref] | 0.39 ^j^ | 1 [Ref] | 0.45 ^j^ | 1 [Ref] | 0.98 ^j^ | 1 [Ref] | 0.79 ^j^ | 1 [Ref] | 0.65 ^j^ |
| Non-white | 56 | 26 | 30 | 1.5 (0.8-2.9) |  | 1.5 (0.8-2.9) |  | 1.1 (0.5-2.3) |  | 0.9 (0.3-2.2) |  | 0.7 (0.3-1.9) |  |
| Prefer not to answer | 4 | 2 | 2 | 0.7 (0.1-6.6) |  | 0.8 (0.1-7.6) |  | 1.1 (0.1-10.8) |  | 2 (0.2-21.2) |  | 2 (0.2-21) |  |
| Education |  |  |  |  |  |  |  |  |  |  |  |  |  |
| No college | 30 | 16 | 14 | 1 [Ref] | 0.66 | 1 [Ref] | 0.89 | 1 [Ref] | 0.04 | 1 [Ref] | 0.30 | 1 [Ref] | 0.52 |
| Some college or 2-yr degree | 108 | 48 | 60 | 0.7 (0.3-1.7) |  | 0.8 (0.3-1.9) |  | 0.3 (0.1-0.8) |  | 0.4 (0.2-1.3) |  | 0.5 (0.2-1.6) |  |
| 4-yr degree or greater | 50 | 30 | 20 | 0.7 (0.3-1.7) |  | 0.9 (0.3-2.3) |  | 0.3 (0.1-0.9) |  | 0.6 (0.2-1.8) |  | 0.7 (0.2-2.3) |  |
| Income (US $) |  |  |  |  |  |  |  |  |  |  |  |  |  |
| <$35K | 58 | 28 | 30 | 1 [Ref] | 0.45 ^j^ | 1 [Ref] | 0.76 ^j^ | 1 [Ref] | 0.16 ^j^ | 1 [Ref] | 0.08 ^j^ | 1 [Ref] | 0.15 ^j^ |
| $35-$75K | 74 | 32 | 42 | 0.7 (0.4-1.5) |  | 0.7 (0.4-1.5) |  | 0.7 (0.3-1.5) |  | 0.3 (0.1-0.9) |  | 0.4 (0.1-1) |  |
| >$75K | 48 | 28 | 20 | 0.6 (0.3-1.3) |  | 0.7 (0.3-1.6) |  | 0.4 (0.1-1) |  | 0.3 (0.1-0.98) |  | 0.4 (0.1-1.1) |  |
| Prefer not to answer | 8 | 6 | 2 | 0.4 (0.1-2.1) |  | 0.5 (0.1-2.7) |  | 0.2 (0-2.1) |  | 0.3 (0.03-3.1) |  | 0.4 (0.04-3.5) |  |
| Employment |  |  |  |  |  |  |  |  |  |  |  |  |  |
| No | 34 | 18 | 16 | 1 [Ref] | 0.24 | 1 [Ref] | 0.34 | 1 [Ref] | 0.27 | 1 [Ref] | 0.11 ^j^ | 1 [Ref] | 0.22 ^j^ |
| Yes, <20 hours per week | 37 | 17 | 20 | 0.4 (0.2-1.2) |  | 0.5 (0.2-1.3) |  | 0.4 (0.1-1.3) |  | 0.3 (0.1-1.3) |  | 0.4 (0.1-1.5) |  |
| Yes, ≥20 hours per week | 117 | 59 | 58 | 0.6 (0.3-1.4) |  | 0.7 (0.3-1.6) |  | 0.6 (0.2-1.3) |  | 0.4 (0.1-0.98) |  | 0.4 (0.2-1.2) |  |
| Self-reported health |  |  |  |  |  |  |  |  |  |  |  |  |  |
| Fair or Poor | 27 | 15 | 12 | 1 [Ref] | 0.31 | 1 [Ref] | 0.46 | 1 [Ref] | 0.82 | 1 [Ref] | 0.52 ^j^ | 1 [Ref] | 0.34 ^j^ |
| Good | 99 | 51 | 48 | 0.7 (0.3-1.6) |  | 1 (0.4-2.7) |  | 0.7 (0.3-1.9) |  | 1.3 (0.3-4.9) |  | 2 (0.4-9.5) |  |
| Very good to Excellent | 62 | 28 | 34 | 1.1 (0.4-2.8) |  | 1.6 (0.6-4.1) |  | 0.8 (0.3-2.3) |  | 1.9 (0.5-7.8) |  | 3 (0.6-15) |  |
| Smartphone type |  |  |  |  |  |  |  |  |  |  |  |  |  |
| Android | 75 | 34 | 41 | 1 [Ref] | 0.81 | 1 [Ref] | 0.26 | 1 [Ref] | 0.46 | 1 [Ref] | 0.78 | 1 [Ref] | 0.66 |
| iPhone | 113 | 60 | 53 | 1.1 (0.6-2) |  | 1.4 (0.8-2.7) |  | 0.8 (0.4-1.5) |  | 0.9 (0.4-2.1) |  | 0.8 (0.4-1.9) |  |
| Smoking and quitting behavior |  |  |  |  |  |  |  |  |  |  |  |  |  |
| Cigarettes smoked per day | 188 | 94 | 94 | 1 (0.9-1) | 0.15 | 1 (0.9-1) | 0.14 | 1 (0.9-1) | 0.13 | 1 (0.9-1) | 0.28 | 1 (0.9-1) | 0.21 |
| Years smoking | 188 | 94 | 94 | 1 (0.9-1) | 0.15 | 1 (0.9-1) | 0.18 | 1 (0.95-1) | 0.30 | 1 (1-1) | 0.77 | 1 (0.95-1) | 0.69 |
| First cigarette smoked after waking |  |  |  |  |  |  |  |  |  |  |  |  |  |
| Within 5 minutes | 67 | 30 | 37 | 1 [Ref] | 0.75 | 1 [Ref] | 0.75 | 1 [Ref] | 0.90 | 1 [Ref] | 0.37^j^ | 1 [Ref] | 0.44^j^ |
| 6 to 30 minutes | 92 | 47 | 45 | 1 (0.5-1.8) |  | 1 (0.5-1.9) |  | 1.2 (0.6-2.5) |  | 0.9 (0.4-2.1) |  | 1 (0.4-2.4) |  |
| After 30 minutes | 29 | 17 | 12 | 0.7 (0.3-1.8) |  | 0.7 (0.3-1.8) |  | 1 (0.4-2.8) |  | 0.3 (0.1-1.6) |  | 0.4 (0.1-1.8) |  |
| Tobacco products used other than cigarettes |  |  |  |  |  |  |  |  |  |  |  |  |  |
| True | 26 | 15 | 11 | 1 [Ref] | 0.54 | 1 [Ref] | 0.30 | 1 [Ref] | 0.24 | 1 [Ref] | 0.23 ^j^ | 1 [Ref] | 0.19 ^j^ |
| False | 162 | 79 | 83 | 0.8 (0.3-1.8) |  | 0.6 (0.3-1.5) |  | 0.6 (0.2-1.4) |  | 0.5 (0.2-1.5) |  | 0.5 (0.2-1.4) |  |
| HSI^d^ | 188 | 94 | 94 | 1 (0.7-1.2) | 0.71 | 0.9 (0.7-1.2) | 0.61 | 0.9 (0.7-1.2) | 0.41 | 1 (0.7-1.4) | 0.93 | 0.9 (0.7-1.3) | 0.68 |
| Quit attempts in the past 12 months | 188 | 94 | 94 | 1 (0.9-1.1) | 0.47 | 1 (1-1.1) | 0.42 | 1.1 (0.99-1.2) | 0.10 | 1 (0.9-1.1) | 0.98 | 1 (0.9-1.1) | 0.98 |
| Methods used in past quit attempts^e^ |  |  |  |  |  |  |  |  |  |  |  |  |  |
| Something |  |  |  |  |  |  |  |  |  |  |  |  |  |
| False | 16 | 5 | 11 | 1 [Ref] | 0.17 | 1 [Ref] | 0.28 | 1 [Ref] | 0.73 | 1 [Ref] | 0.97 | 1 [Ref] | 0.93 |
| True | 172 | 89 | 83 | 0.5 (0.2-1.4) |  | 0.6 (0.2-1.6) |  | 1.3 (0.3-4.7) |  | 1 (0.2-4.7) |  | 0.9 (0.2-4.5) |  |
| Cold Turkey |  |  |  |  |  |  |  |  |  |  |  |  |  |
| False | 48 | 27 | 21 | 1 [Ref] | 0.06 | 1 [Ref] | 0.08 | 1 [Ref] | 0.06 | 1 [Ref] | 0.08 | 1 [Ref] | 0.054 |
| True | 140 | 67 | 73 | 0.5 (0.3-1) |  | 0.5 (0.3-1.1) |  | 0.5 (0.2-1) |  | 0.5 (0.2-1.1) |  | 0.4 (0.2-1) |  |
| NRT^f^ |  |  |  |  |  |  |  |  |  |  |  |  |  |
| False | 96 | 41 | 55 | 1 [Ref] | 0.53 | 1 [Ref] | 0.57 | 1 [Ref] | 0.84 | 1 [Ref] | 0.39 | 1 [Ref] | 0.29 |
| True | 92 | 53 | 39 | 0.8 (0.4-1.5) |  | 0.8 (0.5-1.6) |  | 1.1 (0.5-2.1) |  | 0.7 (0.3-1.6) |  | 0.6 (0.3-1.5) |  |
| e-Cigarettes or vaping |  |  |  |  |  |  |  |  |  |  |  |  |  |
| False | 123 | 61 | 62 | 1 [Ref] | 0.27 | 1 [Ref] | 0.25 | 1 [Ref] | 0.15 | 1 [Ref] | 0.48 | 1 [Ref] | 0.38 |
| True | 65 | 33 | 32 | 1.4 (0.8-2.6) |  | 1.5 (0.8-2.7) |  | 1.7 (0.8-3.3) |  | 1.4 (0.6-3.2) |  | 1.5 (0.6-3.4) |  |
| Varenicline or Bupropion |  |  |  |  |  |  |  |  |  |  |  |  |  |
| False | 117 | 55 | 62 | 1 [Ref] | 0.34 | 1 [Ref] | 0.24 | 1 [Ref] | 0.32 | 1 [Ref] | 0.12 | 1 [Ref] | 0.17 |
| True | 71 | 39 | 32 | 0.7 (0.4-1.4) |  | 0.7 (0.4-1.3) |  | 0.7 (0.3-1.4) |  | 0.5 (0.2-1.2) |  | 0.5 (0.2-1.3) |  |
| Hypnotherapy |  |  |  |  |  |  |  |  |  |  |  |  |  |
| False | 177 | 87 | 90 | 1 [Ref] | 0.24 | 1 [Ref] | 0.40 | 1 [Ref] | 0.14 | 1 [Ref] | 0.29 | 1 [Ref] | 0.26 |
| True | 11 | 7 | 4 | 2.1 (0.6-7.2) |  | 1.7 (0.5-5.8) |  | 2.6 (0.7-9) |  | 2.1 (0.5-8.9) |  | 2.3 (0.5-9.4) |  |
| Attitudes toward quitting smoking |  |  |  |  |  |  |  |  |  |  |  |  |  |
| DTQ^g^ | 188 | 94 | 94 | 1.1 (0.9-1.2) | 0.29 | PV 0.9 (0.7-1.1) QG 1.2 (1-1.4) | 0.02 | 1 (0.9-1.2) | 0.93 | 0.9 (0.8-1.1) | 0.48 | 0.9 (0.7-1.1) | 0.27 |
| STQ^h^ | 188 | 94 | 94 | 1.1 (0.9-1.2) | 0.34 | 1 (0.9-1.2) | 0.46 | 1 (0.9-1.2) | 0.50 | 1 (0.8-1.2) | 0.93 | 1 (0.8-1.1) | 0.63 |
| SASEQ^i^ | 188 | 94 | 94 | 1 (1-1.1) | 0.23 | 1 (1-1.1) | 0.22 | 1 (0.9-1.1) | 0.80 | 1 (0.9-1.1) | 0.75 | 1 (0.9-1.1) | 0.90 |

^a^ For detection of statistically significant interaction, baseline predictor presented within each cohort: Pivot (PV), QuitGuide(QG); otherwise, adjusted results presented

^b^ Odds Ratio (OR), 95% CI and *P* value (*P*) presented

^c^ Reference for categorical data denoted as [Ref]

^d^ HSI: Heaviness of Smoking Index—low (0-1), medium (2-4), and high (5-6).

^e^ Participants were asked to select all that apply.

^f^ NRT: nicotine replacement therapy.

^g^ DTQ: difficulty to stay quit—If you were to quit smoking right now, how difficult do you think it would be to stay smoke free? (1=really hard to stay quit; 10=really easy to stay quit).

^h^ STQ: success to quit—If you were to quit smoking right now, how successful would you be? (1=not at all successful; 10=completely successful).

^i^ SASEQ: Smoking Abstinence Self-Efficacy Questionnaire (score 1-24).

^j^ Logistic regression with Firth bias correction performed on interaction model only due to quasi-complete separation. Joint test *P* value was not significant.
