# Supplementary material for: Long-Term Outcomes of a Comprehensive Mobile Smoking Cessation Program With Nicotine Replacement Therapy in Adult Smokers: Pilot Randomized Controlled Trial
Source: JMIR Mhealth Uhealth. 2023 Sep 18;11:e48157. doi: 10.2196/48157 (PMC10546267; doi:10.2196/48157)
Supplement: Multimedia Appendix 2 [file mhealth_v11i1e48157_app2.pdf]

CONSORT-EHEALTH (V 1.6.1) - Submission/Publication Form

The CONSORT-EHEALTH checklist is intended for authors of randomized trials evaluating web-based and Internet-based applications/interventions, including mobile interventions, electronic games (incl multiplayer games), social media, certain telehealth applications, and other interactive and/or networked electronic applications. Some of the items (e.g. all subitems under item 5 - description of the intervention) may also be applicable for other study designs.

The goal of the CONSORT EHEALTH checklist and guideline is to be
a) a guide for reporting for authors of RCTs,
b) to form a basis for appraisal of an ehealth trial (in terms of validity)

CONSORT-EHEALTH items/subitems are MANDATORY reporting items for studies published in the Journal of Medical Internet Research and other journals / scientific societies endorsing the checklist.

Items numbered 1., 2., 3., 4a., 4b etc are original CONSORT or CONSORT-NPT (non-pharmacologic treatment) items.
Items with Roman numerals (i., ii, iii, iv etc.) are CONSORT-EHEALTH extensions/clarifications.

As the CONSORT-EHEALTH checklist is still considered in a formative stage, we would ask that you also RATE ON A SCALE OF 1-5 how important/useful you feel each item is FOR THE PURPOSE OF THE CHECKLIST and reporting guideline (optional).

Mandatory reporting items are marked with a red *.
In the textboxes, either copy & paste the relevant sections from your manuscript into this form - please include any quotes from your manuscript in QUOTATION MARKS,
or answer directly by providing additional information not in the manuscript, or elaborating on why the item was not relevant for this study.

YOUR ANSWERS WILL BE PUBLISHED AS A SUPPLEMENTARY FILE TO YOUR PUBLICATION IN JMIR AND ARE CONSIDERED PART OF YOUR PUBLICATION (IF ACCEPTED).
Please fill in these questions diligently. Information will not be copyedited, so please use proper spelling and grammar, use correct capitalization, and avoid abbreviations.

DO NOT FORGET TO SAVE AS PDF _AND_ CLICK THE SUBMIT BUTTON SO YOUR ANSWERS ARE IN OUR DATABASE !!!

Citation Suggestion (if you append the pdf as Appendix we suggest to cite this paper in the caption):
Eysenbach G, CONSORT-EHEALTH Group

CONSORT-EHEALTH: Improving and Standardizing Evaluation Reports of Web-based and Mobile Health Interventions
J Med Internet Res 2011;13(4):e126
URL: http://www.jmir.org/2011/4/e126/
doi: 10.2196/jmir.1923
PMID: 22209829

marler@pivot.co Switch account

Not shared

Draft not saved

* Indicates required question

Your name *

First Last

Jennifer Marler

Primary Affiliation (short), City, Country *

University of Toronto, Toronto, Canada

Pivot Health Technologies, Inc.

Your e-mail address *

abc@gmail.com

marler@pivot.co

Title of your manuscript *

Provide the (draft) title of your manuscript.

Long-Term Outcomes of a Comprehensive Mobile Smoking Cessation Program with Nicotine Replacement Therapy in Adult Smokers: Pilot Randomized Controlled Trial

Name of your App/Software/Intervention *

If there is a short and a long/alternate name, write the short name first and add the long name in brackets.

Pivot: Make impactful changes

Evaluated Version (if any)

e.g. "V1", "Release 2017-03-01", "Version 2.0.27913"

Your answer

Language(s) *

What language is the intervention/app in? If multiple languages are available, separate by comma (e.g. "English, French")

English

URL of your Intervention Website or App

e.g. a direct link to the mobile app on app in appstore (itunes, Google Play), or URL of the website. If the intervention is a DVD or hardware, you can also link to an Amazon page.

https://play.google.com/store/apps/details?id=com.carrot.pivot https://pivot.co/ https://a

URL of an image/screenshot (optional)

Your answer

Accessibility *

Can an enduser access the intervention presently?

- ○ access is free and open
- ○ access only for special usergroups, not open
- ◉ access is open to everyone, but requires payment/subscription/in-app purchases
- ○ app/intervention no longer accessible
- ○ Other:

Primary Medical Indication/Disease/Condition *

e.g. "Stress", "Diabetes", or define the target group in brackets after the condition, e.g. "Autism (Parents of children with)", "Alzheimers (Informal Caregivers of)"

Smoking cessation

Primary Outcomes measured in trial *

comma-separated list of primary outcomes reported in the trial

"The primary aim was to compare smoking ces

Secondary/other outcomes

Are there any other outcomes the intervention is expected to affect?

"At the 52-week time point, study outcomes focused on smoking behavior and self-efficacy. Assessments comprised SASEQ, quit attempts, CPD, the use of NRT, and smoking cessation via self-reported 7-day PPA, 30-day PPA and continuous abstinence, biochemically confirmed abstinence, biochemically confirmed continuous abstinence, self-reported abstinence from all tobacco products."

Recommended "Dose" *

What do the instructions for users say on how often the app should be used?

- ○ Approximately Daily
- ○ Approximately Weekly
- ○ Approximately Monthly
- ○ Approximately Yearly
- ◉ "as needed"
- ○ Other:

Approx. Percentage of Users (starters) still using the app as recommended after 3 months *

- (selected) unknown / not evaluated
- 0-10%
- 11-20%
- 21-30%
- 31-40%
- 41-50%
- 51-60%
- 61-70%
- 71%-80%
- 81-90%
- 91-100%
- Other:

Overall, was the app/intervention effective? *

- (selected) yes: all primary outcomes were significantly better in intervention group vs control
- partly: SOME primary outcomes were significantly better in intervention group vs control
- no statistically significant difference between control and intervention
- potentially harmful: control was significantly better than intervention in one or more outcomes
- inconclusive: more research is needed
- Other:

Article Preparation Status/Stage *

At which stage in your article preparation are you currently (at the time you fill in this form)

- ( ) not submitted yet - in early draft status
- ( ) not submitted yet - in late draft status, just before submission
- ( ) submitted to a journal but not reviewed yet
- ( ) submitted to a journal and after receiving initial reviewer comments
- (x) submitted to a journal and accepted, but not published yet
- ( ) published
- ( ) Other:

Journal *

If you already know where you will submit this paper (or if it is already submitted), please provide the journal name (if it is not JMIR, provide the journal name under "other")

- ( ) not submitted yet / unclear where I will submit this
- ( ) Journal of Medical Internet Research (JMIR)
- (x) JMIR mHealth and UHealth
- ( ) JMIR Serious Games
- ( ) JMIR Mental Health
- ( ) JMIR Public Health
- ( ) JMIR Formative Research
- ( ) Other JMIR sister journal
- ( ) Other:

Is this a full powered effectiveness trial or a pilot/feasibility trial? *

- (●) Pilot/feasibility
- ( ) Fully powered

Manuscript tracking number *

If this is a JMIR submission, please provide the manuscript tracking number under "other" (The ms tracking number can be found in the submission acknowledgement email, or when you login as author in JMIR. If the paper is already published in JMIR, then the ms tracking number is the four-digit number at the end of the DOI, to be found at the bottom of each published article in JMIR)

- ( ) no ms number (yet) / not (yet) submitted to / published in JMIR
- (●) Other: ms# 48157

TITLE AND ABSTRACT

1a) TITLE: Identification as a randomized trial in the title

1a) Does your paper address CONSORT item 1a? *

I.e does the title contain the phrase "Randomized Controlled Trial"? (if not, explain the reason under "other")

- (●) yes
- ( ) Other:

1a-i) Identify the mode of delivery in the title

Identify the mode of delivery. Preferably use “web-based” and/or “mobile” and/or “electronic game” in the title. Avoid ambiguous terms like “online”, “virtual”, “interactive”. Use “Internet-based” only if Intervention includes non-web-based Internet components (e.g. email), use “computer-based” or “electronic” only if offline products are used. Use “virtual” only in the context of “virtual reality” (3-D worlds). Use “online” only in the context of “online support groups”. Complement or substitute product names with broader terms for the class of products (such as “mobile” or “smart phone” instead of “iphone”), especially if the application runs on different platforms.

subitem not at all important

1 ○

2 ○

3 ●

4 ○

5 ○

essential

Clear selection

Does your paper address subitem 1a-i? *

Copy and paste relevant sections from manuscript title (include quotes in quotation marks "like this" to indicate direct quotes from your manuscript), or elaborate on this item by providing additional information not in the ms, or briefly explain why the item is not applicable/relevant for your study

We use the term, "...Mobile Smoking Cessation Program..." in the title

1a-ii) Non-web-based components or important co-interventions in title

Mention non-web-based components or important co-interventions in title, if any (e.g., “with telephone support”).

subitem not at all important

- 1 ○
- 2 ○
- 3 ◉
- 4 ○
- 5 ○

essential

Clear selection

Does your paper address subitem 1a-ii?

Copy and paste relevant sections from manuscript title (include quotes in quotation marks "like this" to indicate direct quotes from your manuscript), or elaborate on this item by providing additional information not in the ms, or briefly explain why the item is not applicable/relevant for your study

We use the term, "...with Nicotine Replacement Therapy..." in the title

1a-iii) Primary condition or target group in the title

Mention primary condition or target group in the title, if any (e.g., “for children with Type I Diabetes”) Example: A Web-based and Mobile Intervention with Telephone Support for Children with Type I Diabetes: Randomized Controlled Trial

subitem not at all important

- 1 ○
- 2 ○
- 3 ◉
- 4 ○
- 5 ○

essential

Clear selection

Does your paper address subitem 1a-iii? *

Copy and paste relevant sections from manuscript title (include quotes in quotation marks "like this" to indicate direct quotes from your manuscript), or elaborate on this item by providing additional information not in the ms, or briefly explain why the item is not applicable/relevant for your study

We use the term, "...in Adult Smokers..." in the title

1b) ABSTRACT: Structured summary of trial design, methods, results, and conclusions

NPT extension: Description of experimental treatment, comparator, care providers, centers, and blinding status.

1b-i) Key features/functionalities/components of the intervention and comparator in the METHODS section of the ABSTRACT

Mention key features/functionalities/components of the intervention and comparator in the abstract. If possible, also mention theories and principles used for designing the site. Keep in mind the needs of systematic reviewers and indexers by including important synonyms. (Note: Only report in the abstract what the main paper is reporting. If this information is missing from the main body of text, consider adding it)

subitem not at all important

1 ○

2 ○

3 ○

4 ○

5 ○

essential

Does your paper address subitem 1b-i? *

Copy and paste relevant sections from the manuscript abstract (include quotes in quotation marks "like this" to indicate direct quotes from your manuscript), or elaborate on this item by providing additional information not in the ms, or briefly explain why the item is not applicable/relevant for your study

"The primary aim was to compare smoking cessation outcomes at 52 weeks in adult smokers randomized to a mobile smoking cessation program: Pivot (intervention) versus QuitGuide (control). " This was addressed in more detail in the first paper from this RCT, which reported 6-month outcomes (https://mhealth.jmir.org/2022/11/e41658)

1b-ii) Level of human involvement in the METHODS section of the ABSTRACT

Clarify the level of human involvement in the abstract, e.g., use phrases like “fully automated” vs. “therapist/nurse/care provider/physician-assisted” (mention number and expertise of providers involved, if any). (Note: Only report in the abstract what the main paper is reporting. If this information is missing from the main body of text, consider adding it)

subitem not at all important

1 ○

2 ○

3 ○

4 ○

5 ○

essential

Does your paper address subitem 1b-ii?

Copy and paste relevant sections from the manuscript abstract (include quotes in quotation marks "like this" to indicate direct quotes from your manuscript), or elaborate on this item by providing additional information not in the ms, or briefly explain why the item is not applicable/relevant for your study

We use the phrases, "mobile smoking cessation program", "remote pilot RCT, "cigarette smokers in the U.S. were recruited online", "Data were self-reported via online questionnaire, with videoconference biovalidation in participants who reported 7-day point prevalence abstinence (PPA)."

1b-iii) Open vs. closed, web-based (self-assessment) vs. face-to-face assessments in the METHODS section of the ABSTRACT

Mention how participants were recruited (online vs. offline), e.g., from an open access website or from a clinic or a closed online user group (closed usergroup trial), and clarify if this was a purely web-based trial, or there were face-to-face components (as part of the intervention or for assessment). Clearly say if outcomes were self-assessed through questionnaires (as common in web-based trials). Note: In traditional offline trials, an open trial (open-label trial) is a type of clinical trial in which both the researchers and participants know which treatment is being administered. To avoid confusion, use “blinded” or “unblinded” to indicated the level of blinding instead of “open”, as “open” in web-based trials usually refers to “open access” (i.e. participants can self-enrol). (Note: Only report in the abstract what the main paper is reporting. If this information is missing from the main body of text, consider adding it)

subitem not at all important

1 ○

2 ○

3 ○

4 ○

5 ○

essential

Does your paper address subitem 1b-iii?

Copy and paste relevant sections from the manuscript abstract (include quotes in quotation marks "like this" to indicate direct quotes from your manuscript), or elaborate on this item by providing additional information not in the ms, or briefly explain why the item is not applicable/relevant for your study

We stated, "In this remote pilot RCT, cigarette smokers in the U.S. were recruited online..." and "Data were self-reported via online questionnaire, with videoconference biovalidation in participants who reported 7-day point prevalence abstinence (PPA)." And, "Cessation outcomes included self-reported 7- and 30-day PPA, abstinence from all tobacco products and continuous abstinence. PPA and continuous abstinence were biovalidated via witnessed breath carbon monoxide (CO) samples."

1b-iv) RESULTS section in abstract must contain use data

Report number of participants enrolled/assessed in each group, the use/uptake of the intervention (e.g., attrition/adherence metrics, use over time, number of logins etc.), in addition to primary/secondary outcomes. (Note: Only report in the abstract what the main paper is reporting. If this information is missing from the main body of text, consider adding it)

subitem not at all important

1 ○

2 ○

3 ○

4 ○

5 ○

essential

Does your paper address subitem 1b-iv?

Copy and paste relevant sections from the manuscript abstract (include quotes in quotation marks "like this" to indicate direct quotes from your manuscript), or elaborate on this item by providing additional information not in the ms, or briefly explain why the item is not applicable/relevant for your study

"Participants comprised 188 smokers (94 Pivot, 94 QuitGuide): mean (SD) age 46.4 (9.2) years, 104 women (55.3%), 128 White individuals (68.1%), mean (SD) CPD 17.6 (9.0). Several cessation rates were higher in Pivot (intention to treat, ITT): self-reported continuous abstinence: 20% (19/94) versus QuitGuide 9% (8/94) (P=.03), biochemically confirmed abstinence: 31% (29/94) versus QuitGuide 18% (17/94) (P=.04), and biochemically confirmed continuous abstinence: 19% (18/94) versus QuitGuide 9% (8/94) (P=.046). " The information on use/uptake was published in the first paper, which detailed outcomes through 6 months https://mhealth.jmir.org/2022/11/e41658 The current paper focuses on longer term outcomes.

1b-v) CONCLUSIONS/DISCUSSION in abstract for negative trials

Conclusions/Discussions in abstract for negative trials: Discuss the primary outcome - if the trial is negative (primary outcome not changed), and the intervention was not used, discuss whether negative results are attributable to lack of uptake and discuss reasons. (Note: Only report in the abstract what the main paper is reporting. If this information is missing from the main body of text, consider adding it)

subitem not at all important

1 ○

2 ○

3 ○

4 ○

5 ○

essential

Does your paper address subitem 1b-v?

Copy and paste relevant sections from the manuscript abstract (include quotes in quotation marks "like this" to indicate direct quotes from your manuscript), or elaborate on this item by providing additional information not in the ms, or briefly explain why the item is not applicable/relevant for your study

The trial was not negative. The Conclusions/Discussion reads, "This pilot RCT supports the long-term effectiveness of the Pivot mobile smoking cessation program with abstinence rates durable through 52 weeks."

INTRODUCTION

2a) In INTRODUCTION: Scientific background and explanation of rationale

2a-i) Problem and the type of system/solution

Describe the problem and the type of system/solution that is object of the study: intended as stand-alone intervention vs. incorporated in broader health care program? Intended for a particular patient population? Goals of the intervention, e.g., being more cost-effective to other interventions, replace or complement other solutions? (Note: Details about the intervention are provided in “Methods” under 5)

subitem not at all important

1 ○

2 ○

3 ○

4 ○

5 ○

essential

Does your paper address subitem 2a-i? *

Copy and paste relevant sections from the manuscript (include quotes in quotation marks "like this" to indicate direct quotes from your manuscript), or elaborate on this item by providing additional information not in the ms, or briefly explain why the item is not applicable/relevant for your study

"In 2020, approximately 12.5% of U.S. adults (approximately 30.8 million people) currently smoked cigarettes [1]. While this represents a marked decrease in the peak prevalence of 42.6% in 1964, smoking remains a formidable public health problem as the leading preventable cause of death, illness and disability in the U.S. [2,3]. Behavioral intervention and pharmacotherapy are proven effective smoking cessation tools. Traditionally, behavioral intervention via counseling has been delivered in person in one-on-one or group sessions or remotely through phone quitlines. Food and Drug Administration (FDA)-approved pharmacotherapy such as NRT is available over the counter and by prescription; bupropion and varenicline are available by prescription. Use of combined behavioral support and pharmacotherapy increases the chance of smoking cessation by 70-100% compared to just brief advice or support [4]. Use of monotherapy is also effective, with NRT, bupropion, varenicline and individual counseling increasing the chance of success by 50-60%, 50-80%, 100-140% and 40-80%, respectively, compared to minimal support or placebo [4,5]. Unfortunately, the impact of these interventions is limited; less than one-third of adult cigarette smokers who make a quit attempt use any type of cessation counseling or FDA-approved pharmacotherapy [6]. Most quit attempts are unassisted, with resultant success rates of approximately 7-8% [3]. The rise of mobile smoking cessation interventions seeks to address this shortcoming by leveraging the ubiquitous nature of smartphones; in 2021, 85% of U.S. adults owned smartphones [7]."

2a-ii) Scientific background, rationale: What is known about the (type of) system

Scientific background, rationale: What is known about the (type of) system that is the object of the study (be sure to discuss the use of similar systems for other conditions/diagnoses, if appropiate), motivation for the study, i.e. what are the reasons for and what is the context for this specific study, from which stakeholder viewpoint is the study performed, potential impact of findings [2]. Briefly justify the choice of the comparator.

subitem not at all important

1 ○

2 ○

3 ○

4 ○

5 ○

essential

Does your paper address subitem 2a-ii? *

Copy and paste relevant sections from the manuscript (include quotes in quotation marks "like this" to indicate direct quotes from your manuscript), or elaborate on this item by providing additional information not in the ms, or briefly explain why the item is not applicable/relevant for your study

""Behavioral intervention and pharmacotherapy are proven effective smoking cessation tools. Traditionally, behavioral intervention via counseling has been delivered in person in one-on-one or group sessions or remotely through phone quitlines. Food and Drug Administration (FDA)-approved pharmacotherapy such as NRT is available over the counter and by prescription; bupropion and varenicline are available by prescription...Unfortunately, the impact of these interventions is limited; less than one-third of adult cigarette smokers who make a quit attempt use any type of cessation counseling or FDA-approved pharmacotherapy [6]. Most quit attempts are unassisted, with resultant success rates of approximately 7-8% [3].
The rise of mobile smoking cessation interventions seeks to address this shortcoming by leveraging the ubiquitous nature of smartphones; in 2021, 85% of U.S. adults owned smartphones [7]...we performed a remote pilot RCT in which participants were randomized to Pivot (intervention, an app-based smoking cessation program comprising activities and lessons; a personal CO breath sensor; in-app, text-based human-provided coaching; NRT and a moderated web-based community) or the NCI's QuitGuide app (control). Participants had access to 12 weeks of NRT."

2b) In INTRODUCTION: Specific objectives or hypotheses

Does your paper address CONSORT subitem 2b? *

Copy and paste relevant sections from the manuscript (include quotes in quotation marks "like this" to indicate direct quotes from your manuscript), or elaborate on this item by providing additional information not in the ms, or briefly explain why the item is not applicable/relevant for your study

"Objectives: The primary aim of this study was to assess longer-term smoking cessation outcomes in the aforementioned RCT in which Pivot was compared with QuitGuide [17]. Specifically, we aimed to compare self-reported and biovalidated point prevalence abstinence and continuous abstinence at 52 weeks from enrollment. Secondary aims included assessment of other aspects of smoking-related behavior such as quit attempts, CPD and self-efficacy, and the performance of exploratory analyses of predictors of smoking cessation."

METHODS

3a) Description of trial design (such as parallel, factorial) including allocation ratio

Does your paper address CONSORT subitem 3a? *

Copy and paste relevant sections from the manuscript (include quotes in quotation marks "like this" to indicate direct quotes from your manuscript), or elaborate on this item by providing additional information not in the ms, or briefly explain why the item is not applicable/relevant for your study

"In this two-arm, parallel-group, non-crossover, single-center, individually-randomized controlled trial, participants were randomized to one of two app-based smoking cessation programs: Pivot (intervention) or QuitGuide (control)."

3b) Important changes to methods after trial commencement (such as eligibility criteria), with reasons

Does your paper address CONSORT subitem 3b? *

Copy and paste relevant sections from the manuscript (include quotes in quotation marks "like this" to indicate direct quotes from your manuscript), or elaborate on this item by providing additional information not in the ms, or briefly explain why the item is not applicable/relevant for your study

N/A, there were no important changes to method after trial commencement

3b-i) Bug fixes, Downtimes, Content Changes

Bug fixes, Downtimes, Content Changes: ehealth systems are often dynamic systems. A description of changes to methods therefore also includes important changes made on the intervention or comparator during the trial (e.g., major bug fixes or changes in the functionality or content) (5-iii) and other “unexpected events” that may have influenced study design such as staff changes, system failures/downtimes, etc. [2].

subitem not at all important

1 ○

2 ○

3 ○

4 ○

5 ○

essential

Does your paper address subitem 3b-i?

Copy and paste relevant sections from the manuscript (include quotes in quotation marks "like this" to indicate direct quotes from your manuscript), or elaborate on this item by providing additional information not in the ms, or briefly explain why the item is not applicable/relevant for your study

N/A there were no important changes made on the intervention or comparator during the trial (e.g., major bug fixes or changes in the functionality or content) or other “unexpected events” that may have influenced study design such as staff changes, system failures/downtimes, etc.

4a) Eligibility criteria for participants

Does your paper address CONSORT subitem 4a? *

Copy and paste relevant sections from the manuscript (include quotes in quotation marks "like this" to indicate direct quotes from your manuscript), or elaborate on this item by providing additional information not in the ms, or briefly explain why the item is not applicable/relevant for your study

"Eligibility criteria included: 21+ years of age, current daily cigarette smoker (≥ 5 CPD) for the past 12 months, plans to quit smoking in the next 30 days, resident of the United States, able to read and comprehend English, owns and uses a smartphone compatible with the study app (iPhone 5 and above with operating system iOS 12 and above, or, Android 7.0 and above with operating system Android 7.0 and above), has daily internet access on smartphone, and self-reported comfort with downloading and using smartphone apps."

4a-i) Computer / Internet literacy

Computer / Internet literacy is often an implicit "de facto" eligibility criterion - this should be explicitly clarified.

subitem not at all important

1 ○

2 ○

3 ○

4 ○

5 ○

essential

Does your paper address subitem 4a-i?

Copy and paste relevant sections from the manuscript (include quotes in quotation marks "like this" to indicate direct quotes from your manuscript), or elaborate on this item by providing additional information not in the ms, or briefly explain why the item is not applicable/relevant for your study

Participants had, "...self-reported comfort with downloading and using smartphone

4a-ii) Open vs. closed, web-based vs. face-to-face assessments:

Open vs. closed, web-based vs. face-to-face assessments: Mention how participants were recruited (online vs. offline), e.g., from an open access website or from a clinic, and clarify if this was a purely web-based trial, or there were face-to-face components (as part of the intervention or for assessment), i.e., to what degree got the study team to know the participant. In online-only trials, clarify if participants were quasi-anonymous and whether having multiple identities was possible or whether technical or logistical measures (e.g., cookies, email confirmation, phone calls) were used to detect/prevent these.

subitem not at all important

1 ○

2 ○

3 ○

4 ○

5 ○

essential

Does your paper address subitem 4a-ii? *

Copy and paste relevant sections from the manuscript (include quotes in quotation marks "like this" to indicate direct quotes from your manuscript), or elaborate on this item by providing additional information not in the ms, or briefly explain why the item is not applicable/relevant for your study

"Participants were recruited in the United States through web media (Facebook and Google Ads). Potential participants were asked to provide contact information and answer questions on demographics using an online screening form. Study staff reviewed each online screening form...During the screening phone call, potential participants were asked questions to confirm study eligibility. During this call, study personnel informed the potential participant of the study details and answered any questions. Potential eligible participants who wanted to proceed with the study were emailed an electronic HIPAA Authorization form and an electronic Informed Consent Form, which they signed before participating in this study...Biovalidation was sought at 52 weeks in individuals who reported 7-day (or greater) PPA on the associated questionnaire. A video call with study staff and the participant was scheduled within 7 days following the participant's response to the associated questionnaire...Participants who indicated they were not at least 7 days abstinent, and/or that they smoke ≥ 1 CPD were not eligible to undergo further biovalidation testing during the visit. Participants who indicated they were at least 7 days abstinent and do not smoke cigarettes were eligible to proceed with the testing. Participants who indicated they had smoked any other combustible materials over the previous 24 hours were eligible to undergo biovalidation test at that same visit, with the possibility of scheduling a follow-up biovalidation test for the following day with instruction to not smoke the previously reported other combustible substance(s) over the intervening 24-hour period...Biovalidation was obtained through CO breath sampling...Data collection was performed via online questionnaires at baseline and at the 52-week follow-up..."

4a-iii) Information giving during recruitment

Information given during recruitment. Specify how participants were briefed for recruitment and in the informed consent procedures (e.g., publish the informed consent documentation as appendix, see also item X26), as this information may have an effect on user self-selection, user expectation and may also bias results.

subitem not at all important

1 ○

2 ○

3 ○

4 ○

5 ○

essential

Does your paper address subitem 4a-iii?

Copy and paste relevant sections from the manuscript (include quotes in quotation marks "like this" to indicate direct quotes from your manuscript), or elaborate on this item by providing additional information not in the ms, or briefly explain why the item is not applicable/relevant for your study

All participants underwent an informative screening phone call. "During the screening phone call, potential participants were asked questions to confirm study eligibility. During this call, study personnel informed the potential participant of the study details and answered any questions. Potential eligible participants who wanted to proceed with the study were emailed an electronic HIPAA Authorization form and an electronic Informed Consent Form, which they signed before participating in this study."
Participants were read a standardized script during the screening/information phone call.
This script was approved by the IRB. Potential participants were aware of the possible program functions they may or may not have access to during the study: in-app text based
coaching, access to an online moderated community forum and a personal carbon monoxide breath sensor. Potential participants were aware all study participants would have
access to an app-based cessation program and up to 12 weeks of free nicotine replacement

4b) Settings and locations where the data were collected

Does your paper address CONSORT subitem 4b? *

Copy and paste relevant sections from the manuscript (include quotes in quotation marks "like this" to indicate direct quotes from your manuscript), or elaborate on this item by providing additional information not in the ms, or briefly explain why the item is not applicable/relevant for your study

"Data collection was performed via online questionnaires at baseline and at the 52-week follow-up. Study data were imported directly into a secure database (PostgreSQL, PostgreSQL Global Development Group)."
"Biovalidation was obtained through CO breath sampling. Participants in the intervention arm used their Pivot Breath Sensor for this test. Shortly before the visit, participants in the control arm were mailed a Pivot Breath Sensor limited to 10 breath samples. On the video call, participants held the breath sensor up to the screen immediately after completing the breath sample so that study staff could see and record the CO ppm measurement on the sensor screen."

4b-i) Report if outcomes were (self-)assessed through online questionnaires

Clearly report if outcomes were (self-)assessed through online questionnaires (as common in web-based trials) or otherwise.

subitem not at all important

1 ○

2 ○

3 ○

4 ○

5 ○

essential

Does your paper address subitem 4b-i? *

Copy and paste relevant sections from the manuscript (include quotes in quotation marks "like this" to indicate direct quotes from your manuscript), or elaborate on this item by providing additional information not in the ms, or briefly explain why the item is not applicable/relevant for your study

"Data collection was performed via online questionnaires at baseline and at the 52-week follow-up...Participants were considered to have achieved self-reported 7-day (30-day) PPA if they answered 'no' to the following question: 'In the last 7 (30) days have you smoked any cigarettes, even a single puff?'. Biochemically confirmed abstinence was defined as self-reporting 7-day abstinence and a breath CO sample <10 ppm at the 52-week biovalidation visit. Self-reported continuous abstinence was defined as self-report of 7-day (or greater) PPA at 12 weeks, self-report of 30-day PPA at 26 weeks and 52 weeks, no more than 5 cigarettes between 12 weeks and 26 weeks, and 0 cigarettes between 26 weeks and 52 weeks. Biochemically confirmed continuous abstinence was defined as self-reported continuous abstinence as detailed above, with a breath CO sample <10 ppm at each of the associated 12-, 26- and 52-week biovalidation visits. Abstinence from all tobacco products was self-reported."

4b-ii) Report how institutional affiliations are displayed

Report how institutional affiliations are displayed to potential participants [on ehealth media], as affiliations with prestigious hospitals or universities may affect volunteer rates, use, and reactions with regards to an intervention.(Not a required item – describe only if this may bias results)

subitem not at all important

1 ○

2 ○

3 ○

4 ○

5 ○

essential

Does your paper address subitem 4b-ii?

Copy and paste relevant sections from the manuscript (include quotes in quotation marks "like this" to indicate direct quotes from your manuscript), or elaborate on this item by providing additional information not in the ms, or briefly explain why the item is not applicable/relevant for your study

Your answer

5) The interventions for each group with sufficient details to allow replication, including how and when they were actually administered

5-i) Mention names, credential, affiliations of the developers, sponsors, and owners

Mention names, credential, affiliations of the developers, sponsors, and owners [6] (if authors/evaluators are owners or developer of the software, this needs to be declared in a “Conflict of interest” section or mentioned elsewhere in the manuscript).

subitem not at all important

1 ○

2 ○

3 ○

4 ○

5 ○

essential

Does your paper address subitem 5-i?

Copy and paste relevant sections from the manuscript (include quotes in quotation marks "like this" to indicate direct quotes from your manuscript), or elaborate on this item by providing additional information not in the ms, or briefly explain why the item is not applicable/relevant for your study

"Conflicts of Interest
JM, CF, MU, DB, and DU are current employees of Pivot Health Technologies Inc. (Pivot), the developer of the Pivot smoking cessation program. They receive salary and stock options from Pivot. DU is the President and CEO of Pivot and an investor in the company. JG is a paid statistical consultant."

5-ii) Describe the history/development process

Describe the history/development process of the application and previous formative evaluations (e.g., focus groups, usability testing), as these will have an impact on adoption/use rates and help with interpreting results.

subitem not at all important

1 ○

2 ○

3 ○

4 ○

5 ○

essential

Does your paper address subitem 5-ii?

Copy and paste relevant sections from the manuscript (include quotes in quotation marks "like this" to indicate direct quotes from your manuscript), or elaborate on this item by providing additional information not in the ms, or briefly explain why the item is not applicable/relevant for your study

From the first paper published on this RCT (https://mhealth.jmir.org/2022/11/e41658/): "A cohort study of the Pivot program was published in 2021. During the study, Pivot included a mobile app, personal CO breath sensor, and text-based human coaching. At 3 months post-program completion (mean 7.2 months after enrollment), 32.0% (ITT) and 37.5% (Completer) of participants achieved 7-day PPA; 27.6% (ITT) and 32.4% (Completer) reported 30-day PPA [15]. The Pivot program has since undergone updates, and now includes access to NRT and a moderated online community. These changes, the need for long-term results for app-based cessation programs, and the ongoing need to assess the performance of Pivot within the context of current smoking cessation programs, warrant new investigation of the Pivot program..."
From the current manuscipt, "we performed a remote pilot RCT in which participants were randomized to Pivot (intervention, an app-based smoking cessation program comprising activities and lessons; a personal CO breath sensor; in-app, text-based human-provided coaching; NRT and a moderated web-based community) or the NCI's QuitGuide app (control). Participants had access to 12 weeks of NRT. At 6 months, 34/94 (36.2%) of Pivot participants and 25/94 (26.6%) of QuitGuide participants self-reported 7-day PPA (ITT, RR 1.3 95% CI 0.8 to 1.9; P=.27), and 26/94 (27.7%) of Pivot participants and 14/94 (14.9%) of QuitGuide participants achieved biovalidated abstinence (ITT, RR 1.9 95% CI 1.1 to 3.5; P=.02) [17]. In the context of these RCTs, the natural next step in the evolution of data pertaining to mobile smoking cessation programs is the establishment of longer-term outcomes through smoking cessation durability."

5-iii) Revisions and updating

Revisions and updating. Clearly mention the date and/or version number of the application/intervention (and comparator, if applicable) evaluated, or describe whether the intervention underwent major changes during the evaluation process, or whether the development and/or content was “frozen” during the trial. Describe dynamic components such as news feeds or changing content which may have an impact on the replicability of the intervention (for unexpected events see item 3b).

subitem not at all important

1 ○

2 ○

3 ○

4 ○

5 ○

essential

Does your paper address subitem 5-iii?

Copy and paste relevant sections from the manuscript (include quotes in quotation marks "like this" to indicate direct quotes from your manuscript), or elaborate on this item by providing additional information not in the ms, or briefly explain why the item is not applicable/relevant for your study

The intervention and comparator did not undergo major changes during the evaluation process.

5-iv) Quality assurance methods

Provide information on quality assurance methods to ensure accuracy and quality of information provided [1], if applicable.

subitem not at all important

1 ○

2 ○

3 ○

4 ○

5 ○

essential

Does your paper address subitem 5-iv?

Copy and paste relevant sections from the manuscript (include quotes in quotation marks "like this" to indicate direct quotes from your manuscript), or elaborate on this item by providing additional information not in the ms, or briefly explain why the item is not applicable/relevant for your study

Your answer

5-v) Ensure replicability by publishing the source code, and/or providing screenshots/screen-capture video, and/or providing flowcharts of the algorithms used

Ensure replicability by publishing the source code, and/or providing screenshots/screen-capture video, and/or providing flowcharts of the algorithms used. Replicability (i.e., other researchers should in principle be able to replicate the study) is a hallmark of scientific reporting.

subitem not at all important

1 ○

2 ○

3 ○

4 ○

5 ○

essential

Does your paper address subitem 5-v?

Copy and paste relevant sections from the manuscript (include quotes in quotation marks "like this" to indicate direct quotes from your manuscript), or elaborate on this item by providing additional information not in the ms, or briefly explain why the item is not applicable/relevant for your study

Screenshots are provided as a Multi-media appendix in the first paper published on this RCT: https://mhealth.jmir.org/2022/11/e41658/

5-vi) Digital preservation

Digital preservation: Provide the URL of the application, but as the intervention is likely to change or disappear over the course of the years; also make sure the intervention is archived (Internet Archive, webcitation.org, and/or publishing the source code or screenshots/videos alongside the article). As pages behind login screens cannot be archived, consider creating demo pages which are accessible without login.

subitem not at all important

1 ○

2 ○

3 ○

4 ○

5 ○

essential

Does your paper address subitem 5-vi?

Copy and paste relevant sections from the manuscript (include quotes in quotation marks "like this" to indicate direct quotes from your manuscript), or elaborate on this item by providing additional information not in the ms, or briefly explain why the item is not applicable/relevant for your study

https://play.google.com/store/apps/details?id=com.carrot.pivot https://pivot.co/ https://apps.apple.com/us/app/pivot-make-impactful-changes/id1236893700

5-vii) Access

Access: Describe how participants accessed the application, in what setting/context, if they had to pay (or were paid) or not, whether they had to be a member of specific group. If known, describe how participants obtained "access to the platform and Internet" [1]. To ensure access for editors/reviewers/readers, consider to provide a "backdoor" login account or demo mode for reviewers/readers to explore the application (also important for archiving purposes, see vi).

subitem not at all important

1 ○

2 ○

3 ○

4 ○

5 ○

essential

Does your paper address subitem 5-vii? *

Copy and paste relevant sections from the manuscript (include quotes in quotation marks "like this" to indicate direct quotes from your manuscript), or elaborate on this item by providing additional information not in the ms, or briefly explain why the item is not applicable/relevant for your study

Participants were emailed a link with log-on code (if applicable) for their respective app to
get started. They did not have to pay to access the app. They were not paid to access the app.

5-viii) Mode of delivery, features/functionalities/components of the intervention and comparator, and the theoretical framework

Describe mode of delivery, features/functionalities/components of the intervention and comparator, and the theoretical framework [6] used to design them (instructional strategy [1], behaviour change techniques, persuasive features, etc., see e.g., [7, 8] for terminology). This includes an in-depth description of the content (including where it is coming from and who developed it) [1],” whether [and how] it is tailored to individual circumstances and allows users to track their progress and receive feedback” [6]. This also includes a description of communication delivery channels and – if computer-mediated communication is a component – whether communication was synchronous or asynchronous [6]. It also includes information on presentation strategies [1], including page design principles, average amount of text on pages, presence of hyperlinks to other resources, etc. [1].

subitem not at all important

1 ○

2 ○

3 ○

4 ○

5 ○

essential

Does your paper address subitem 5-viii? *

Copy and paste relevant sections from the manuscript (include quotes in quotation marks "like this" to indicate direct quotes from your manuscript), or elaborate on this item by providing additional information not in the ms, or briefly explain why the item is not applicable/relevant for your study

" Intervention: Pivot
Pivot is a 12-month digital smoking cessation program based on the USCPG for tobacco cessation. Pivot includes the Pivot Breath Sensor and Pivot app (Pivot Health Technologies Inc., San Carlos, CA).
The Pivot Breath Sensor is a portable, personal mobile breath sensor that measures the level of CO in exhaled breath. The user submits a breath sample by exhaling into the sensor mouthpiece. The sensor displays the exhaled breath CO value in parts per million (ppm) to the user directly on the device. When paired to the user's smartphone, the user's CO values also populate the user's Pivot app. Displayed CO values are color coded and categorized as most consistent with not smoking (green, 0-6 ppm), possibly smoking (orange, 7-9 ppm) or smoking (red, ≥ 10 ppm).
The self-guided Pivot app leverages evidence-based principles and clinical best practices. This includes the USCPG-recommended 5 As, tailoring on readiness to quit [4], the provision of FDA-approved NRT with accompanying education on use and adherence [4,26-27], the incorporation of effective methods for smoking cessation based on cognitive behavioral therapy (CBT) and self-determination theory [3,28-29] and CBT-based counseling through a live, dedicated coach [3-4,30]. Pivot app functions include interactive educational activities, the ability to log cigarettes, set a quit date, create a quit plan, complete practice quits (1-24 hours in duration), play educational games, watch educational videos, interact with one's dedicated human coach via in-app text messaging, view CO breath sample values and trends, learn about and then order NRT, access the moderated online Pivot community discussion forum, share goals and progress with the online Pivot community discussion forum or one's social network via text messaging or email, and complete daily check-ins after quit date.
The educational journey in the Pivot App comprises 4 tracts: Learn, Reduce, Prepare to Quit, and Maintain My Quit, and is designed to accommodate smokers along the spectrum of readiness to quit. Participants may navigate between tracts as desired, to access content most relevant to their goals and needs.
Pivot users are assigned a human coach with whom they work one-on-one over the duration of their use of Pivot (up to one year). Communication between coach and Pivot user is via asynchronous in-app text messaging. Pivot coaches are tobacco treatment specialists. The coach reaches out periodically, approximately once per week, during the participant's active use of Pivot. Participants may reach out to their coach whenever and however often they like.
Pivot users may access the moderated online discussion community through the Pivot app. The forum is moderated by a tobacco treatment specialist. The online community forum is a place to give and receive support and advice from others going through the Pivot program.

Control: QuitGuide
QuitGuide is a product of Smokefree.gov [31] a smoking cessation resource created by the Tobacco Control Research Branch at the NCI in collaboration with tobacco control professionals and smoking cessation experts, and with input from ex-smokers [32]. A well-established smoking cessation app, QuitGuide has been used in previous RCTs in which digital smoking cessation programs were compared [14-15,33]. The app focuses on helping users understand their smoking patterns and build the skills needed to become and stay smoke-free [32]. Specifically, QuitGuide helps users: focus on motivations to quit; prepare to quit through developing a quit plan, identifying and planning how to address triggers and moods, teaching about FDA-approved smoking cessation medications, and identifying and [illegible]

providing access to social support; quit smoking by acknowledging user progress and teaching skills to address cravings; and stay quit by presenting tips and motivations to stay smoke-free and address slips if they occur. QuitGuide app functions include educational reading activities, including focus on FDA-approved cessation medications and associated adherence. Additional QuitGuide app functions comprise tracking and reviewing cigarettes, moods, triggers and cravings, setting tip message notifications for locations and times when one is prone to smoke, setting a quit date, creating a quit plan, completing journal entries, sharing goals and progress with one's social network via text messaging or email, accessing additional chat and phone support, and providing updates on quit status after quit date.
QuitGuide was used as the control for the following reasons: the content follows the USCPG for tobacco cessation; it is an app-based smoking cessation program, thereby enabling intrastudy comparison of same-modality interventions; the app is nonproprietary and is free to the public; and its use in previous well-designed RCTs [14-15,33] provides context and enables interstudy comparison to earlier data."

5-ix) Describe use parameters

Describe use parameters (e.g., intended "doses" and optimal timing for use). Clarify what instructions or recommendations were given to the user, e.g., regarding timing, frequency, heaviness of use, if any, or was the intervention used ad libitum.

subitem not at all important

1 ○

2 ○

3 ○

4 ○

5 ○

essential

Does your paper address subitem 5-ix?

Copy and paste relevant sections from the manuscript (include quotes in quotation marks "like this" to indicate direct quotes from your manuscript), or elaborate on this item by providing additional information not in the ms, or briefly explain why the item is not applicable/relevant for your study

During the screening call, participants were told that suggested use of the breath sensor (should they receive one) was 4 times a day, spread out over the course of the day, acknowledging they should ultimately use the sensor how it best suits their life. They were also told, "We suggest you use all components of your assigned quit smoking program during the study." No other use recommendations were made.

5-x) Clarify the level of human involvement

Clarify the level of human involvement (care providers or health professionals, also technical assistance) in the e-intervention or as co-intervention (detail number and expertise of professionals involved, if any, as well as “type of assistance offered, the timing and frequency of the support, how it is initiated, and the medium by which the assistance is delivered”. It may be necessary to distinguish between the level of human involvement required for the trial, and the level of human involvement required for a routine application outside of a RCT setting (discuss under item 21 – generalizability).

subitem not at all important

1 ○

2 ○

3 ○

4 ○

5 ○

essential

Does your paper address subitem 5-x?

Copy and paste relevant sections from the manuscript (include quotes in quotation marks "like this" to indicate direct quotes from your manuscript), or elaborate on this item by providing additional information not in the ms, or briefly explain why the item is not applicable/relevant for your study

"Pivot users are assigned a human coach with whom they work one-on-one over the duration of their use of Pivot (up to one year). Communication between coach and Pivot user is via asynchronous in-app text messaging. Pivot coaches are tobacco treatment specialists. The coach reaches out periodically, approximately once per week, during the participant's active use of Pivot. Participants may reach out to their coach whenever and however often they like.
Pivot users may access the moderated online discussion community through the Pivot app. The forum is moderated by a tobacco treatment specialist. The online community forum is a place to give and receive support and advice from others going through the Pivot program."

5-xi) Report any prompts/reminders used

Report any prompts/reminders used: Clarify if there were prompts (letters, emails, phone calls, SMS) to use the application, what triggered them, frequency etc. It may be necessary to distinguish between the level of prompts/reminders required for the trial, and the level of prompts/reminders for a routine application outside of a RCT setting (discuss under item 21 – generalizability).

subitem not at all important

1 ○

2 ○

3 ○

4 ○

5 ○

essential

Does your paper address subitem 5-xi? *

Copy and paste relevant sections from the manuscript (include quotes in quotation marks "like this" to indicate direct quotes from your manuscript), or elaborate on this item by providing additional information not in the ms, or briefly explain why the item is not applicable/relevant for your study

From the first paper on this RCT (https://mhealth.jmir.org/2022/11/e41658/): "A total of 6 reminders to prompt use of the program were emailed to all participants every other week over the first 12 weeks of the study." For the second part of the study reported on in the current manuscript, participants were not provided any prompts for use of their assigned program.

5-xii) Describe any co-interventions (incl. training/support)

Describe any co-interventions (incl. training/support): Clearly state any interventions that are provided in addition to the targeted eHealth intervention, as ehealth intervention may not be designed as stand-alone intervention. This includes training sessions and support [1]. It may be necessary to distinguish between the level of training required for the trial, and the level of training for a routine application outside of a RCT setting (discuss under item 21 – generalizability.

subitem not at all important

1 ○

2 ○

3 ○

4 ○

5 ○

essential

Does your paper address subitem 5-xii? *

Copy and paste relevant sections from the manuscript (include quotes in quotation marks "like this" to indicate direct quotes from your manuscript), or elaborate on this item by providing additional information not in the ms, or briefly explain why the item is not applicable/relevant for your study

No additional training/interventions were required of study participants. The eHealth interventions (Pivot and QuitGuide) were designed as stand-alone interventions. Study training staff trained on how to conduct the biovalidation visits, which were videocalls during which they read a standardized script to participants.

6a) Completely defined pre-specified primary and secondary outcome measures, including how and when they were assessed

Does your paper address CONSORT subitem 6a? *

Copy and paste relevant sections from the manuscript (include quotes in quotation marks "like this" to indicate direct quotes from your manuscript), or elaborate on this item by providing additional information not in the ms, or briefly explain why the item is not applicable/relevant for your study

"Baseline
The following variables were collected at baseline: demographic information (age, gender, race/ethnicity, household income, education, employment status, smartphone type), smoking status, smoking history, Heaviness of Smoking Index (HSI) [36], success to quit (STQ, scale 1-10), difficulty to stay quit (DTQ, scale 1-10) [37-38], and Smoking Abstinence Self-efficacy Questionnaire (SASEQ), a 6-item survey describing emotional or social situations for which smokers indicate on a 5-point Likert scale (0–4) whether they will be able to refrain from smoking, with total higher scores representing higher self-efficacy [39].
52 Weeks
At the 52-week time point, study outcomes focused on smoking behavior and self-efficacy. Assessments comprised SASEQ, quit attempts, CPD, the use of NRT, and smoking cessation via self-reported 7-day PPA, 30-day PPA and continuous abstinence, biochemically confirmed abstinence, biochemically confirmed continuous abstinence, self-reported abstinence from all tobacco products.
Self-efficacy was assessed through the SASEQ. Participants were considered to have made a quit attempt during the study if they answered ≥1 to the following question: “Since you began the study, how many times have you tried to quit smoking where you've gone at least 1 day without smoking a cigarette, even a single puff?”. From this question, mean (SD) quit attempts per participant were quantified as well. CPD were assessed through mean percentage change and the proportion of participants who reduced their CPD by ≥ 50% compared to baseline. NRT use included whether a participant ordered NRT (yes or no), and if so, the type of NRT they ordered, using participant-placed orders.
Participants were considered to have achieved self-reported 7-day (30-day) PPA if they answered “no” to the following question: “In the last 7 (30) days have you smoked any cigarettes, even a single puff?”. Biochemically confirmed abstinence was defined as self-reporting 7-day abstinence and a breath CO sample <10 ppm at the 52-week biovalidation visit. Self-reported continuous abstinence was defined as self-report of 7-day (or greater) PPA at 12 weeks, self-report of 30-day PPA at 26 weeks and 52 weeks, no more than 5 cigarettes between 12 weeks and 26 weeks, and 0 cigarettes between 26 weeks and 52 weeks. Biochemically confirmed continuous abstinence was defined as self-reported continuous abstinence as detailed above, with a breath CO sample <10 ppm at each of the associated 12-, 26- and 52-week biovalidation visits. Abstinence from all tobacco products was self-reported."

6a-i) Online questionnaires: describe if they were validated for online use and apply CHERRIES items to describe how the questionnaires were designed/deployed

If outcomes were obtained through online questionnaires, describe if they were validated for online use and apply CHERRIES items to describe how the questionnaires were designed/deployed [9].

subitem not at all important

1 ○

2 ○

3 ○

4 ○

5 ○

essential

Does your paper address subitem 6a-i?

Copy and paste relevant sections from manuscript text

Your answer

6a-ii) Describe whether and how “use” (including intensity of use/dosage) was defined/measured/monitored

Describe whether and how “use” (including intensity of use/dosage) was defined/measured/monitored (logins, logfile analysis, etc.). Use/adoption metrics are important process outcomes that should be reported in any ehealth trial.

subitem not at all important

1 ○

2 ○

3 ○

4 ○

5 ○

essential

Does your paper address subitem 6a-ii?

Copy and paste relevant sections from manuscript text

See https://mhealth.jmir.org/2022/11/e41658/
Participants were asked the following questions weekly for the first 12 weeks of the study:
1. Approximately how many days did you open your study app over the last 7 days? Select
one: □ 0 □ 1 □ 2 □ 3 □ 4 □ 5 □ 6 □ 7
2. Approximately how many times have you opened your study app over the last 7 days?

The 6-12 month period reported on in the present manuscript was focused on long-term cessation outcomes after this follow-up period; use of the program was not

6a-iii) Describe whether, how, and when qualitative feedback from participants was obtained

Describe whether, how, and when qualitative feedback from participants was obtained (e.g., through emails, feedback forms, interviews, focus groups).

subitem not at all important

1 ○

2 ○

3 ○

4 ○

5 ○

essential

Does your paper address subitem 6a-iii?

Copy and paste relevant sections from manuscript text

Qualitative feedback was obtained from the online questionnaires participants completed.
Examples of qualitative questions asked include, 'How would you improve the experience of
your assigned program?' 'What has been the most helpful part of your program?' 'What has
been the most frustrating part of your program?' Participant responses to these questions will be addressed elsewhere, as they are outside the scope of the current

6b) Any changes to trial outcomes after the trial commenced, with reasons

Does your paper address CONSORT subitem 6b? *

Copy and paste relevant sections from the manuscript (include quotes in quotation marks "like this" to indicate direct quotes from your manuscript), or elaborate on this item by providing additional information not in the ms, or briefly explain why the item is not applicable/relevant for your study

There were no changes to trial outcomes after the trial commenced.

7a) How sample size was determined

NPT: When applicable, details of whether and how the clustering by care provides or centers was addressed

7a-i) Describe whether and how expected attrition was taken into account when calculating the sample size

Describe whether and how expected attrition was taken into account when calculating the sample size.

subitem not at all important

1 ○

2 ○

3 ○

4 ○

5 ○

essential

Does your paper address subitem 7a-i?

Copy and paste relevant sections from manuscript title (include quotes in quotation marks "like this" to indicate direct quotes from your manuscript), or elaborate on this item by providing additional information not in the ms, or briefly explain why the item is not applicable/relevant for your study

See https://mhealth.jmir.org/2022/11/e41658/:
"As this is a pilot RCT and the first assessment of Pivot compared to usual care, the sample
size is powered to show differences in engagement, specifically, the number of times participants opened their assigned app over the first 12 weeks of the study. In previous clinical studies, Pivot mean app openings were 24.2-38.7 (SD range 20.8-25.9) by 90 days
(data on file). In addition, Bricker et al. reported app openings comparing ACT-based smoking cessation apps (SmartQuit and iCanQuit) to QuitGuide. In one study, at 2-month follow-up, Bricker et al. reported mean (SD) app openings were 37.2 (46.1) for SmartQuit and 15.2 (13.6) for QuitGuide [5]. In a subsequent study, at 12-month follow-up, mean (SD)
app openings were 37.5 (88.4) for iCanQuit and 9.9 (50.0) for QuitGuide [4].
Based on this data, we estimated mean (SD) 25 (25) app openings in the Pivot intervention
arm vs. 15 (19) app openings in the QuitGuide control arm at 12 weeks. Detecting a difference of 10 app openings between Pivot and QuitGuide with 0.8 power and 0.05 alpha
would require 156.7 participants, which we round up to 158. In a previous study, 272/319 (85.3%) participants completed the end-of-Pivot questionnaire at a mean (SD) 4.1 (1.4) months after enrollment [3743]. In assessing the primary endpoint at 3 months (12 weeks),
we included an expected 15% attrition rate, with the aim to enroll up to 180 participants (up

7b) When applicable, explanation of any interim analyses and stopping guidelines

Does your paper address CONSORT subitem 7b? *

Copy and paste relevant sections from the manuscript (include quotes in quotation marks "like this" to indicate direct quotes from your manuscript), or elaborate on this item by providing additional information not in the ms, or briefly explain why the item is not applicable/relevant for your study

Explanation of interim analyses and stopping guidelines is not applicable.

8a) Method used to generate the random allocation sequence
NPT: When applicable, how care providers were allocated to each trial group

Does your paper address CONSORT subitem 8a? *

Copy and paste relevant sections from the manuscript (include quotes in quotation marks "like this" to indicate direct quotes from your manuscript), or elaborate on this item by providing additional information not in the ms, or briefly explain why the item is not applicable/relevant for your study

"The allocation sequence was provided by Study Randomizer software application (2017) [18]."

8b) Type of randomisation; details of any restriction (such as blocking and block size)

Does your paper address CONSORT subitem 8b? *

Copy and paste relevant sections from the manuscript (include quotes in quotation marks "like this" to indicate direct quotes from your manuscript), or elaborate on this item by providing additional information not in the ms, or briefly explain why the item is not applicable/relevant for your study

"Participants were randomly assigned in a computer-generated 1:1 ratio to either QuitGuide or Pivot using randomly permuted blocks of size 2 and 4."

9) Mechanism used to implement the random allocation sequence (such as sequentially numbered containers), describing any steps taken to conceal the sequence until interventions were assigned

Does your paper address CONSORT subitem 9? *

Copy and paste relevant sections from the manuscript (include quotes in quotation marks "like this" to indicate direct quotes from your manuscript), or elaborate on this item by providing additional information not in the ms, or briefly explain why the item is not applicable/relevant for your study

"The allocation sequence was provided by Study Randomizer software application (2017) [18]...Researchers were blinded to treatment allocation until after randomization was performed."

10) Who generated the random allocation sequence, who enrolled participants, and who assigned participants to interventions

Does your paper address CONSORT subitem 10? *

Copy and paste relevant sections from the manuscript (include quotes in quotation marks "like this" to indicate direct quotes from your manuscript), or elaborate on this item by providing additional information not in the ms, or briefly explain why the item is not applicable/relevant for your study

The Study Randomizer software application generated the random allocation sequence and assigned participants to interventions. Participants were considered enrolled after completing the following 3 tasks: electronically signing the informed consent form, completing the Baseline Questionnaire and reporting the date they first logged in to their assigned app. Study personnel designated participants as enrolled after they completed these 3 criteria.

11a) If done, who was blinded after assignment to interventions (for example, participants, care providers, those assessing outcomes) and how

NPT: Whether or not administering co-interventions were blinded to group assignment

11a-i) Specify who was blinded, and who wasn't

Specify who was blinded, and who wasn't. Usually, in web-based trials it is not possible to blind the participants [1, 3] (this should be clearly acknowledged), but it may be possible to blind outcome assessors, those doing data analysis or those administering co-interventions (if any).

subitem not at all important

1 ○

2 ○

3 ○

4 ○

5 ○

essential

Does your paper address subitem 11a-i? *

Copy and paste relevant sections from the manuscript (include quotes in quotation marks "like this" to indicate direct quotes from your manuscript), or elaborate on this item by providing additional information not in the ms, or briefly explain why the item is not applicable/relevant for your study

"Researchers were blinded to treatment allocation until after randomization was performed." As it was not possible in this web-based trial, participants were not blinded to treatment allocation

11a-ii) Discuss e.g., whether participants knew which intervention was the "intervention of interest" and which one was the "comparator"

Informed consent procedures (4a-ii) can create biases and certain expectations - discuss e.g., whether participants knew which intervention was the "intervention of interest" and which one was the "comparator".

subitem not at all important

1 ○

2 ○

3 ○

4 ○

5 ○

essential

Does your paper address subitem 11a-ii?

Copy and paste relevant sections from the manuscript (include quotes in quotation marks "like this" to indicate direct quotes from your manuscript), or elaborate on this item by providing additional information not in the ms, or briefly explain why the item is not applicable/relevant for your study

During the informed consent process, participants were informed of possible program features they may or may not have, such as a carbon monoxide breath sensor, app-based human coaching, and access to a moderated online community forum. Participants were not informed of specific characteristics of the two programs (for example, that one program was Pivot and comprised features A-E, and the other program was QuitGuide and comprised features A-B).

11b) If relevant, description of the similarity of interventions

(this item is usually not relevant for ehealth trials as it refers to similarity of a placebo or sham intervention to a active medication/intervention)

Does your paper address CONSORT subitem 11b? *

Copy and paste relevant sections from the manuscript (include quotes in quotation marks "like this" to indicate direct quotes from your manuscript), or elaborate on this item by providing additional information not in the ms, or briefly explain why the item is not applicable/relevant for your study

The similarity of interventions is addressed in the manuscript text included in 5-viii

12a) Statistical methods used to compare groups for primary and secondary outcomes

NPT: When applicable, details of whether and how the clustering by care providers or centers was addressed

Does your paper address CONSORT subitem 12a? *

Copy and paste relevant sections from the manuscript (include quotes in quotation marks "like this" to indicate direct quotes from your manuscript), or elaborate on this item by providing additional information not in the ms, or briefly explain why the item is not applicable/relevant for your study

"For results where a change from baseline can be measured (CPD, SASEQ), each participant’s baseline data served as their control to then calculate a difference at 52 weeks, and a paired 2-tailed t test was used to test for a significant difference from 0. Outcomes were evaluated using regression analyses adjusted for the four randomization stratification covariates to detect differences between the treatment and control arms. Linear regression was used for numerical data to obtain a point estimate of the mean difference. For count outcomes, the IRR was estimated using Poisson regression when the variance to mean ratio was close to 1, or using negative binomial regressions when the variance to mean ratio was >1. For binary outcomes, the OR was estimated using logistic regression, and the RR was estimated using either log-link binomial regression or log-link Poisson regression with robust estimators [40]. For binary outcomes where there was a very high frequency response (e.g., ≥95%), only the RR was presented. For multicategory outcomes of ≥3, multinomial logistic regression was used to test for proportion differences between the study arms.
In the assessment of quit rates (self-reported and biovalidated PPA and continuous abstinence, and self-reported abstinence from all tobacco products), 2 sets of analyses were performed. In the ITT analysis, individuals who did not respond to PPA questions were assumed to be smoking. A study responder analysis was also performed, which only included individuals who completed the 52-week questionnaire. For the outcomes of quit attempts and the proportion who reduced CPD by ≥ 50%, a study responder analysis was performed."

12a-i) Imputation techniques to deal with attrition / missing values

Imputation techniques to deal with attrition / missing values: Not all participants will use the intervention/comparator as intended and attrition is typically high in ehealth trials. Specify how participants who did not use the application or dropped out from the trial were treated in the statistical analysis (a complete case analysis is strongly discouraged, and simple imputation techniques such as LOCF may also be problematic [4]).

subitem not at all important

1 ○

2 ○

3 ○

4 ○

5 ○

essential

Does your paper address subitem 12a-i? *

Copy and paste relevant sections from the manuscript (include quotes in quotation marks "like this" to indicate direct quotes from your manuscript), or elaborate on this item by providing additional information not in the ms, or briefly explain why the item is not applicable/relevant for your study

"In the assessment of quit rates (self-reported and biovalidated PPA and continuous abstinence, and self-reported abstinence from all tobacco products), 2 sets of analyses were performed. In the ITT analysis, individuals who did not respond to PPA questions were assumed to be smoking. A study responder analysis was also performed, which only included individuals who completed the 52-week questionnaire."

From the first paper from this RCT (https://mhealth.jmir.org/2022/11/e41658/):
"The primary end point of the total number of app openings through 12 weeks was calculated by summing the number of weekly app openings, which were reported by the participants weekly and represented total app openings over the preceding 7 days. There were 170 participants who completed all 12 surveys. There were 4 participants (2 in Pivot and 2 in QuitGuide) who withdrew consent by week 3, accounting for 41 incomplete surveys. App openings for these participants were set to 0, as they did not participate in the study. This left 14 participants (8 Pivot and 6 QuitGuide) with one or more surveys not completed for 44 incomplete surveys. Although this only represented 7.4% of total participants and 2% of total surveys, imputation was necessary to calculate the total app openings, total days with app openings, and total weeks with app openings.

There was no pattern of missingness upon visual inspection, and multiple imputation method was performed using SAS multiple imputation procedure full conditional specification predicted mean matching with 25 imputations [46]. The primary end point of total app openings by the intervention and control arms was then compared in a negative binomial regression model adjusted for the 4 randomization covariates in each of the imputations with SAS MIANALYZE. Similarly, total days with app openings and total weeks with app openings were analyzed using negative binomial regression and Poisson regression, respectively. The mean of the imputed data was used for reporting descriptive statistics."

12b) Methods for additional analyses, such as subgroup analyses and adjusted analyses

Does your paper address CONSORT subitem 12b? *

Copy and paste relevant sections from the manuscript (include quotes in quotation marks "like this" to indicate direct quotes from your manuscript), or elaborate on this item by providing additional information not in the ms, or briefly explain why the item is not applicable/relevant for your study

"Predictors: We also performed exploratory post-hoc analyses that may help inform the design of future studies. We used logistic regression to examine associations between baseline characteristics and smoking behavior outcomes at 52 weeks. Each independent baseline variable was evaluated as a predictor in either one or two types of models using the ITT and responder datasets. The first model evaluated the interaction of the independent baseline variable with the randomized cohort. The interaction was tested for significance compared to a model without the interaction using the joint test. If the interaction was significant the results are reported. If not, then the second model without the interaction was applied. In this model, the independent baseline variable was adjusted for the randomization cohort and utilized a Type 3 test for detection of statistical significance. One P value is presented, either from the Joint Test, if significant, or from the Type 3 test. For statistically significant models with baseline characteristics with 3 or more categories, additional permutations of the reference were completed to appropriately characterize the difference.
This evaluation was completed for the binary outcomes of self-reported 7-day PPA and 30-day PPA, biovalidated 7-day PPA, self-reported continuous abstinence and biovalidated continuous abstinence. In contrast to the analysis of outcomes, these models were not adjusted with the four randomization variables. Certain categorical baseline variables were collapsed due to either insufficient tallies for model convergence or to simplify the model evaluation. These baseline variables include ethnicity, education, income, health, use of non-cigarette tobacco products, past quit methods used, and first cigarette smoked after waking. In addition, some of the interaction models produced quasi-complete separation and the data was modeled again with logistic regression using a Firth bias correction [41]. Analyses were conducted using SAS Version 9.4 (SAS Institute, Cary, NC). Statistical significance was set at P<.05."

X26) REB/IRB Approval and Ethical Considerations [recommended as subheading under "Methods"] (not a CONSORT item)

X26-i) Comment on ethics committee approval

subitem not at all important

1 ○

2 ○

3 ○

4 ○

5 ○

essential

Does your paper address subitem X26-i?

Copy and paste relevant sections from the manuscript (include quotes in quotation marks "like this" to indicate direct quotes from your manuscript), or elaborate on this item by providing additional information not in the ms, or briefly explain why the item is not applicable/relevant for your study

"The study was reviewed and approved by the Solutions IRB, LLC (Yarnell, AZ, USA) protocol number 2021/04/38."

x26-ii) Outline informed consent procedures

Outline informed consent procedures e.g., if consent was obtained offline or online (how? Checkbox, etc.?), and what information was provided (see 4a-ii). See [6] for some items to be included in informed consent documents.

subitem not at all important

1 ○

2 ○

3 ○

4 ○

5 ○

essential

Does your paper address subitem X26-ii?

Copy and paste relevant sections from the manuscript (include quotes in quotation marks "like this" to indicate direct quotes from your manuscript), or elaborate on this item by providing additional information not in the ms, or briefly explain why the item is not applicable/relevant for your study

"Potential eligible participants who wanted to proceed with the study were emailed an electronic HIPAA Authorization form and an electronic Informed Consent Form, which they signed before participating in this study."

X26-iii) Safety and security procedures

Safety and security procedures, incl. privacy considerations, and any steps taken to reduce the likelihood or detection of harm (e.g., education and training, availability of a hotline)

subitem not at all important

1 ○

2 ○

3 ○

4 ○

5 ○

essential

Does your paper address subitem X26-iii?

Copy and paste relevant sections from the manuscript (include quotes in quotation marks "like this" to indicate direct quotes from your manuscript), or elaborate on this item by providing additional information not in the ms, or briefly explain why the item is not applicable/relevant for your study

Study team members had documentation of HIPAA / HITECH Security Awareness training
and NIH Protecting Human Research Participants training. The study team takes careful measures to prevent a data breach including use of encryptions, secure connections, and limited access to data. The study team uses appropriate safeguards and complies, where
applicable, with 45 C.F.R. Part 164, Subpart C with respect to Electronic Protected Health Information, to prevent use or disclosure of Protected Health Information. Specifically, Pivot
Health Technologies Inc. has implemented the administrative, physical, and technical safeguards set forth in 45 C.F.R. §§ 164.308, 164.310, and 164.312 that reasonably and appropriately protect the confidentiality, integrity, and availability of any Protected Health Information that it creates, receives, maintains, or transmits. The QuitGuide app is maintained by the U.S. Government. It is protected by various provisions of Title 18, U.S. Code. The QuitGuide app notes, “NCI complies with requirements for privacy and security established by the Office of Management and Budget (OMB), Department of Health and

RESULTS

13a) For each group, the numbers of participants who were randomly assigned, received intended treatment, and were analysed for the primary outcome
NPT: The number of care providers or centers performing the intervention in each group and the number of patients treated by each care provider in each center

Does your paper address CONSORT subitem 13a? *

Copy and paste relevant sections from the manuscript (include quotes in quotation marks "like this" to indicate direct quotes from your manuscript), or elaborate on this item by providing additional information not in the ms, or briefly explain why the item is not applicable/relevant for your study

"From June 2021 to October 2021, a total of 3042 web-based screening forms were received; 533 (17.5%) met the screening eligibility criteria and responded to an initial outbound phone call from study staff. Of these 533 individuals, 188 (35.3%) were randomized and completed enrollment (94 in each arm), comprising the ITT sample (188 randomized/3042 screened = 6.2%; 188 randomized/533 eligible = 35.3%)."

13b) For each group, losses and exclusions after randomisation, together with reasons

Does your paper address CONSORT subitem 13b? (NOTE: Preferably, this is shown in a CONSORT flow diagram) *

Copy and paste relevant sections from the manuscript (include quotes in quotation marks "like this" to indicate direct quotes from your manuscript), or elaborate on this item by providing additional information not in the ms, or briefly explain why the item is not applicable/relevant for your study

"In each arm, 2 participants withdrew consent within the first 3 weeks of the study." This is addressed in the CONSORT flow diagram, which is "Figure 1 Study participant CONSORT (Consolidated Standards of Reporting Trials) flow diagram."

13b-i) Attrition diagram

Strongly recommended: An attrition diagram (e.g., proportion of participants still logging in or using the intervention/comparator in each group plotted over time, similar to a survival curve) or other figures or tables demonstrating usage/dose/engagement.

subitem not at all important

1 ○

2 ○

3 ○

4 ○

5 ○

essential

Does your paper address subitem 13b-i?

Copy and paste relevant sections from the manuscript or cite the figure number if applicable (include quotes in quotation marks "like this" to indicate direct quotes from your manuscript), or elaborate on this item by providing additional information not in the ms, or briefly explain why the item is not applicable/relevant for your study

We elected to not include an attrition diagram and instead included the following text in the first manuscript from this RCT (https://mhealth.jmir.org/2022/11/e41658/), "Self-report of logging into their app at least once a week was reported in ≥ 85% of participants in each arm for each week through 12 weeks; in QuitGuide it ranged from 85% to 97% and in Pivot it was 86% to 98%.
Attrition that occurred later in the study is represented in the current manuscript in the CONSORT diagram ("Figure 1 Study participant CONSORT (Consolidated Standards of Reporting Trials) flow diagram.")
Retention for data collection was high, "The 52-week study questionnaire was completed by 179/188 (95.2%) participants, specifically by 89/94 (94.7%) in the Pivot arm and 90/94 (95.7%) in the QuitGuide arm; these comprise the study responder samples."

14a) Dates defining the periods of recruitment and follow-up

Does your paper address CONSORT subitem 14a? *

Copy and paste relevant sections from the manuscript (include quotes in quotation marks "like this" to indicate direct quotes from your manuscript), or elaborate on this item by providing additional information not in the ms, or briefly explain why the item is not applicable/relevant for your study

"From June to October 2021, 3042 online screening forms were received; 533 met the screening eligibility criteria and responded to an initial outbound phone call from study staff." The last 12-month questionnaire was completed October 30, 2022.

14a-i) Indicate if critical “secular events” fell into the study period

Indicate if critical “secular events” fell into the study period, e.g., significant changes in Internet resources available or “changes in computer hardware or Internet delivery resources”

subitem not at all important

1 ○

2 ○

3 ○

4 ○

5 ○

essential

Does your paper address subitem 14a-i?

Copy and paste relevant sections from the manuscript (include quotes in quotation marks "like this" to indicate direct quotes from your manuscript), or elaborate on this item by providing additional information not in the ms, or briefly explain why the item is not applicable/relevant for your study

No critical secular events fell into the study period.

14b) Why the trial ended or was stopped (early)

Does your paper address CONSORT subitem 14b? *

Copy and paste relevant sections from the manuscript (include quotes in quotation marks "like this" to indicate direct quotes from your manuscript), or elaborate on this item by providing additional information not in the ms, or briefly explain why the item is not applicable/relevant for your study

12-month data collection is complete. This is a two-year study and is ongoing. The trial has not ended and was not stopped early.

15) A table showing baseline demographic and clinical characteristics for each group

NPT: When applicable, a description of care providers (case volume, qualification, expertise, etc.) and centers (volume) in each group

Does your paper address CONSORT subitem 15? *

Copy and paste relevant sections from the manuscript (include quotes in quotation marks "like this" to indicate direct quotes from your manuscript), or elaborate on this item by providing additional information not in the ms, or briefly explain why the item is not applicable/relevant for your study

See "Table 1. Participant baseline data (N=188)"

15-i) Report demographics associated with digital divide issues

In ehealth trials it is particularly important to report demographics associated with digital divide issues, such as age, education, gender, social-economic status, computer/Internet/ehealth literacy of the participants, if known.

subitem not at all important

1 ○

2 ○

3 ○

4 ○

5 ○

essential

Does your paper address subitem 15-i? *

Copy and paste relevant sections from the manuscript (include quotes in quotation marks "like this" to indicate direct quotes from your manuscript), or elaborate on this item by providing additional information not in the ms, or briefly explain why the item is not applicable/relevant for your study

Yes demographics associated with digital divide issues are reported; see "Table 1. Participant baseline data (N=188)"

16) For each group, number of participants (denominator) included in each analysis and whether the analysis was by original assigned groups

16-i) Report multiple “denominators” and provide definitions

Report multiple “denominators” and provide definitions: Report N’s (and effect sizes) “across a range of study participation [and use] thresholds” [1], e.g., N exposed, N consented, N used more than x times, N used more than y weeks, N participants “used” the intervention/comparator at specific pre-defined time points of interest (in absolute and relative numbers per group). Always clearly define “use” of the intervention.

subitem not at all important

1 ○

2 ○

3 ○

4 ○

5 ○

essential

Does your paper address subitem 16-i? *

Copy and paste relevant sections from the manuscript (include quotes in quotation marks "like this" to indicate direct quotes from your manuscript), or elaborate on this item by providing additional information not in the ms, or briefly explain why the item is not applicable/relevant for your study

Yes all results and tables include denominators across a range of study participation and thresholds, noting that attrition was low, so denominators for enrolled and completer analyses were close.

16-ii) Primary analysis should be intent-to-treat

Primary analysis should be intent-to-treat, secondary analyses could include comparing only “users”, with the appropriate caveats that this is no longer a randomized sample (see 18-i).

subitem not at all important

1 ○

2 ○

3 ○

4 ○

5 ○

essential

Does your paper address subitem 16-ii?

Copy and paste relevant sections from the manuscript (include quotes in quotation marks "like this" to indicate direct quotes from your manuscript), or elaborate on this item by providing additional information not in the ms, or briefly explain why the item is not applicable/relevant for your study

Primary analysis is intent-to-treat, see "Table 3. Smoking cessation rates at 26 and 52 weeks (N=188)"

17a) For each primary and secondary outcome, results for each group, and the estimated effect size and its precision (such as 95% confidence interval)

Does your paper address CONSORT subitem 17a? *

Copy and paste relevant sections from the manuscript (include quotes in quotation marks "like this" to indicate direct quotes from your manuscript), or elaborate on this item by providing additional information not in the ms, or briefly explain why the item is not applicable/relevant for your study

For each primary and secondary outcome, results are presented for each group, along with the estimated effect size and its precision. See "Table 3. Smoking cessation rates at 26 and 52 weeks (N=188)"

17a-i) Presentation of process outcomes such as metrics of use and intensity of use

In addition to primary/secondary (clinical) outcomes, the presentation of process outcomes such as metrics of use and intensity of use (dose, exposure) and their operational definitions is critical. This does not only refer to metrics of attrition (13-b) (often a binary variable), but also to more continuous exposure metrics such as “average session length”. These must be accompanied by a technical description how a metric like a “session” is defined (e.g., timeout after idle time) [1] (report under item 6a).

subitem not at all important

1 ○

2 ○

3 ○

4 ○

5 ○

essential

Does your paper address subitem 17a-i?

Copy and paste relevant sections from the manuscript (include quotes in quotation marks "like this" to indicate direct quotes from your manuscript), or elaborate on this item by providing additional information not in the ms, or briefly explain why the item is not applicable/relevant for your study

This seems to pertain to data from the first paper from this RCT. In that instance, our metrics of use included self-reported app openings, days with app openings and weeks with app opening through week 12. Because we did not have access to the QuitGuide use analytic data, we were unable to assess continuous exposure metrics such as average session length.

17b) For binary outcomes, presentation of both absolute and relative effect sizes is recommended

Does your paper address CONSORT subitem 17b? *

Copy and paste relevant sections from the manuscript (include quotes in quotation marks "like this" to indicate direct quotes from your manuscript), or elaborate on this item by providing additional information not in the ms, or briefly explain why the item is not applicable/relevant for your study

In "Table 3. Smoking cessation rates at 26 and 52 weeks (N=188)", odds ratio and relative risk are presented with associated P values.

18) Results of any other analyses performed, including subgroup analyses and adjusted analyses, distinguishing pre-specified from exploratory

Does your paper address CONSORT subitem 18? *

Copy and paste relevant sections from the manuscript (include quotes in quotation marks "like this" to indicate direct quotes from your manuscript), or elaborate on this item by providing additional information not in the ms, or briefly explain why the item is not applicable/relevant for your study

" We performed exploratory post-hoc analyses using logistic regressions to explore qualitative associations between baseline characteristics and smoking behavior outcomes (Multimedia Appendix 1). We identified three variables with statistically significant correlation: age, education and DTQ. None of these variables were considered predictive across all five smoking cessation outcomes."

18-i) Subgroup analysis of comparing only users

A subgroup analysis of comparing only users is not uncommon in ehealth trials, but if done, it must be stressed that this is a self-selected sample and no longer an unbiased sample from a randomized trial (see 16-iii).

subitem not at all important

1 ○

2 ○

3 ○

4 ○

5 ○

essential

Does your paper address subitem 18-i?

Copy and paste relevant sections from the manuscript (include quotes in quotation marks "like this" to indicate direct quotes from your manuscript), or elaborate on this item by providing additional information not in the ms, or briefly explain why the item is not applicable/relevant for your study

We did not do a subgroup analysis comparing only users.

19) All important harms or unintended effects in each group
(for specific guidance see CONSORT for harms)

Does your paper address CONSORT subitem 19? *

Copy and paste relevant sections from the manuscript (include quotes in quotation marks "like this" to indicate direct quotes from your manuscript), or elaborate on this item by providing additional information not in the ms, or briefly explain why the item is not applicable/relevant for your study

There were no important harms or unintended effects in either group.

19-i) Include privacy breaches, technical problems

Include privacy breaches, technical problems. This does not only include physical “harm” to participants, but also incidents such as perceived or real privacy breaches [1], technical problems, and other unexpected/unintended incidents. “Unintended effects” also includes unintended positive effects [2].

subitem not at all important

1 ○

2 ○

3 ○

4 ○

5 ○

essential

Does your paper address subitem 19-i?

Copy and paste relevant sections from the manuscript (include quotes in quotation marks "like this" to indicate direct quotes from your manuscript), or elaborate on this item by providing additional information not in the ms, or briefly explain why the item is not applicable/relevant for your study

There were no privacy breaches or technical problems, including incidents such as perceived or real privacy breaches or other unexpected/unintended incidents.

19-ii) Include qualitative feedback from participants or observations from staff/researchers

Include qualitative feedback from participants or observations from staff/researchers, if available, on strengths and shortcomings of the application, especially if they point to unintended/unexpected effects or uses. This includes (if available) reasons for why people did or did not use the application as intended by the developers.

subitem not at all important

1 ○

2 ○

3 ○

4 ○

5 ○

essential

Does your paper address subitem 19-ii?

Copy and paste relevant sections from the manuscript (include quotes in quotation marks "like this" to indicate direct quotes from your manuscript), or elaborate on this item by providing additional information not in the ms, or briefly explain why the item is not applicable/relevant for your study

While descriptive categorical data was collected from participants and is included in the manuscript (see Results, section titled "Participant Feedback"), qualitative data is outside the scope of the current manuscript.

DISCUSSION

22) Interpretation consistent with results, balancing benefits and harms, and considering other relevant evidence

NPT: In addition, take into account the choice of the comparator, lack of or partial blinding, and unequal expertise of care providers or centers in each group

22-i) Restate study questions and summarize the answers suggested by the data, starting with primary outcomes and process outcomes (use)

Restate study questions and summarize the answers suggested by the data, starting with primary outcomes and process outcomes (use).

subitem not at all important

1 ○

2 ○

3 ○

4 ○

5 ○

essential

Does your paper address subitem 22-i? *

Copy and paste relevant sections from the manuscript (include quotes in quotation marks "like this" to indicate direct quotes from your manuscript), or elaborate on this item by providing additional information not in the ms, or briefly explain why the item is not applicable/relevant for your study

"Here we report results at 52 weeks in this pilot RCT comparing the Pivot and QuitGuide mobile smoking cessation programs among 188 adult cigarette smokers in the U.S. Pivot had higher smoking cessation rates as follows: self-reported continuous abstinence: Pivot 20% (19/94) versus QuitGuide 9% (8/94) (P=.03), biochemically confirmed abstinence: Pivot 31% (29/94) versus QuitGuide 18% (17/94) (P=.04), and biochemically confirmed continuous abstinence: Pivot 19% (18/94) versus QuitGuide 9% (8/94) (P=.046). Pivot participants also had a significant increase in self-efficacy, via SASEQ, whereas the QuitGuide participants did not. And more Pivot participants (99%, 93/94 in Pivot versus 85%, 80/94 in QuitGuide, P<.001) placed more NRT orders: Pivot mean 3.3 (SD 2.0) versus QuitGuide mean 1.8 (SD 1.6) (P<.001).
On average, QuitGuide participants made more quit attempts: Pivot 7.0 (SD 6.3) versus QuitGuide 9.5 (SD 7.5) (P=.01). Among the subset of participants who did not report 7-day (or greater) PPA at 52 weeks, QuitGuide participants had a greater reduction in CPD: Pivot, -34.6% versus QuitGuide, −46.1% (P=.04). Among those who reported 7-day PPA at 52 weeks, the majority in each arm (90%) indicated their study program helped them quit smoking."

22-ii) Highlight unanswered new questions, suggest future research

Highlight unanswered new questions, suggest future research.

subitem not at all important

1 ○

2 ○

3 ○

4 ○

5 ○

essential

Does your paper address subitem 22-ii?

Copy and paste relevant sections from the manuscript (include quotes in quotation marks "like this" to indicate direct quotes from your manuscript), or elaborate on this item by providing additional information not in the ms, or briefly explain why the item is not applicable/relevant for your study

"While beyond the scope of this study, a cost-benefit analysis is an appropriate and logical next step."
"This study compares the two programs but cannot determine the specific effect of these additional tools. Accordingly, appropriate focus of future assessment includes if and to what extent these tools contribute to Pivot outcomes."

20) Trial limitations, addressing sources of potential bias, imprecision, and, if relevant, multiplicity of analyses

20-i) Typical limitations in ehealth trials

Typical limitations in ehealth trials: Participants in ehealth trials are rarely blinded. Ehealth trials often look at a multiplicity of outcomes, increasing risk for a Type I error. Discuss biases due to non-use of the intervention/usability issues, biases through informed consent procedures, unexpected events.

subitem not at all important

1 ○

2 ○

3 ○

4 ○

5 ○

essential

Does your paper address subitem 20-i? *

Copy and paste relevant sections from the manuscript (include quotes in quotation marks "like this" to indicate direct quotes from your manuscript), or elaborate on this item by providing additional information not in the ms, or briefly explain why the item is not applicable/relevant for your study

"This study also had several limitations. First, as a pilot study, this study was not powered for cessation outcomes. Self-reported PPA and abstinence from all tobacco products outcomes were not significantly different between the two study groups, whereas biovalidated abstinence and self-reported continuous abstinence outcomes were. Whether a larger study powered for these outcomes would have resulted in significant differences in smoking abstinence outcomes is unknown.

Second, the Pivot program includes the following additional tools not included in the QuitGuide program: a CO breath sensor, SMS text messaging-based coaching with a tobacco cessation coach, and a moderated web-based community support forum. This study compares the two programs but cannot determine the specific effect of these additional tools. Accordingly, appropriate focus of future assessment includes if and to what extent these tools contribute to Pivot outcomes.

Third, we cannot rule out some influence on participant selection by the recruiting and enrollment process, and how this might influence the generalizability of study outcomes to the general population of smokers. We took steps to mitigate factors that might incentivize enrollment such as the availability of free NRT and study compensation. Specifically, these study characteristics were not mentioned in the recruiting ads, online screening form or until later in the screening phone calls. Potential participants were deemed ineligible if they were already using NRT or other smoking cessation tools at study entry. Steps were also taken to minimize the possible influence of compensation, including not tying compensation to outcome or program use, delaying payments 2 to 3 weeks after the completion of compensated events, and keeping payment amounts conservative. Recruitment was conducted on social media throughout the U.S. and ultimately, study participants represented 42 of the 50 states along with the District of Columbia. We also employed non-proportional quota sampling for several participant characteristics (age group, gender, race, employment status) with potential participants called on a first-come first served basis accordingly. All non-proportional quota sampling goals were met. Nonetheless, it is always important to consider factors that may influence the generalizability of study outcomes to larger populations.

Fourth, after randomization, all researchers were unblinded to participant group allocation, which can result in unbalanced participant communication and data collection efforts. To mitigate this, the study design included scheduled, standardized, and scripted participant communications (written and verbal) reviewed by the IRB. High and comparable questionnaire and biovalidation visit completion rates (>92% for questionnaires and >69% for biovalidation visits at 12, 26, and 52 weeks in both study arms) reflect favorably on our attempt to minimize these possible effects.

Fifth, exhaled CO as a biovalidation test for smoking cessation is imperfect. The half-life of CO is, on average, 4 hours and is influenced by activity level (i.e., shorter half-life when exercising and longer when sleeping). Accordingly, smokers may be able to abstain from smoking for several hours before providing a breath sample and obtain a CO value consistent with "not smoking"; we cannot exclude this occurrence in the biovalidation visits. Moreover, secondhand smoke, use of other combustible substances such as marijuana, and environmental or occupational CO exposure can increase CO levels. That said, the limitations of other biovalidation methods made exhaled CO, which is noninvasive, less expensive, and easy for a lay user to perform the preferred option. Specifically, although cotinine, a nicotine metabolite, has a longer half-life (≥8-30 hours) than CO and therefore requires longer abstinence periods (2-7 days) to reach "nonsmoking" levels, its collection from body fluids is more onerous and will yield positive results in individuals using NRT, [illegible]

which was problematic with our study design. Anabasine and anatabine are minor tobacco alkaloids that are specific for tobacco-derived products (e.g., cigarettes, cigars, and smokeless tobacco). They are well suited for testing individuals for tobacco use who are using NRT. However, these biomarkers require urine collection and chromatography–mass spectrometry measurement [65]. Altogether, when considering the remote nature of this study and the provision of NRT, we felt exhaled CO, despite its imperfections, was the best option for biovalidation.
Finally, this study does not address the reported differential cessation outcomes in the context of cost-effectiveness. While beyond the scope of this study, a cost-benefit analysis is an appropriate and logical next step. Resource use is an important consideration in the assessment of mobile tobacco cessation interventions. We have sought to establish foundational outcomes in Pivot; these inputs should be included in future cost-benefit assessment."

21) Generalisability (external validity, applicability) of the trial findings

NPT: External validity of the trial findings according to the intervention, comparators, patients, and care providers or centers involved in the trial

21-i) Generalizability to other populations

Generalizability to other populations: In particular, discuss generalizability to a general Internet population, outside of a RCT setting, and general patient population, including applicability of the study results for other organizations

subitem not at all important

1 ○

2 ○

3 ○

4 ○

5 ○

essential

Does your paper address subitem 21-i?

Copy and paste relevant sections from the manuscript (include quotes in quotation marks "like this" to indicate direct quotes from your manuscript), or elaborate on this item by providing additional information not in the ms, or briefly explain why the item is not applicable/relevant for your study

"Third, we cannot rule out some influence on participant selection by the recruiting and enrollment process, and how this might influence the generalizability of study outcomes to the general population of smokers. We took steps to mitigate factors that might incentivize enrollment such as the availability of free NRT and study compensation. Specifically, these study characteristics were not mentioned in the recruiting ads, online screening form or until later in the screening phone calls. Potential participants were deemed ineligible if they were already using NRT or other smoking cessation tools at study entry. Steps were also taken to minimize the possible influence of compensation, including not tying compensation to outcome or program use, delaying payments 2 to 3 weeks after the completion of compensated events, and keeping payment amounts conservative. Recruitment was conducted on social media throughout the U.S. and ultimately, study participants represented 42 of the 50 states along with the District of Columbia. We also employed non-proportional quota sampling for several participant characteristics (age group, gender, race, employment status) with potential participants called on a first-come first served basis accordingly. All non-proportional quota sampling goals were met. Nonetheless, it is always important to consider factors that may influence the generalizability of study outcomes to larger populations."

21-ii) Discuss if there were elements in the RCT that would be different in a routine application setting

Discuss if there were elements in the RCT that would be different in a routine application setting (e.g., prompts/reminders, more human involvement, training sessions or other co-interventions) and what impact the omission of these elements could have on use, adoption, or outcomes if the intervention is applied outside of a RCT setting.

subitem not at all important

1 ○

2 ○

3 ○

4 ○

5 ○

essential

Does your paper address subitem 21-ii?

Copy and paste relevant sections from the manuscript (include quotes in quotation marks "like this" to indicate direct quotes from your manuscript), or elaborate on this item by providing additional information not in the ms, or briefly explain why the item is not applicable/relevant for your study

In a routine application setting, there would not be the videoconference biovalidation visits
and the emailed online questionnaires. There were also more human-derived prompts in this
study (6 reminder emails sent every other week from weeks 1-12 from the clinical team email) that would not be present in a non-study setting. And there would not be the screening phone call with study staff to enter the study. Overall, while human contact was
low in this study, it was greater than what is present in a non-study setting, which could have
increased adoption/use of the programs than might be seen in a routine application

OTHER INFORMATION

23) Registration number and name of trial registry

Does your paper address CONSORT subitem 23? *

Copy and paste relevant sections from the manuscript (include quotes in quotation marks "like this" to indicate direct quotes from your manuscript), or elaborate on this item by providing additional information not in the ms, or briefly explain why the item is not applicable/relevant for your study

Clinicaltrials.gov NCT04955639; https://clinicaltrials.gov/ct2/show/NCT04955639

24) Where the full trial protocol can be accessed, if available

Does your paper address CONSORT subitem 24? *

Cite a Multimedia Appendix, other reference, or copy and paste relevant sections from the manuscript (include quotes in quotation marks "like this" to indicate direct quotes from your manuscript), or elaborate on this item by providing additional information not in the ms, or briefly explain why the item is not applicable/relevant for your study

The full protocol is not published but a summary of the protocol is available at Clinicaltrials.gov NCT04955639; https://clinicaltrials.gov/ct2/show/NCT04955639

25) Sources of funding and other support (such as supply of drugs), role of funders

Does your paper address CONSORT subitem 25? *

Copy and paste relevant sections from the manuscript (include quotes in quotation marks "like this" to indicate direct quotes from your manuscript), or elaborate on this item by providing additional information not in the ms, or briefly explain why the item is not applicable/relevant for your study

The informed consent form notes:
"Funding:
This study is funded by Pivot Health Technologies Inc. (formerly Carrot Inc.). Pivot Health Technologies Inc. is the company that has developed one of the programs used in this study. Pivot Health Technologies Inc. develops products to help people quit smoking.
You

X27) Conflicts of Interest (not a CONSORT item)

X27-i) State the relation of the study team towards the system being evaluated

In addition to the usual declaration of interests (financial or otherwise), also state the relation of the study team towards the system being evaluated, i.e., state if the authors/evaluators are distinct from or identical with the developers/sponsors of the intervention.

subitem not at all important

1 ○

2 ○

3 ○

4 ○

5 ○

essential

Does your paper address subitem X27-i?

Copy and paste relevant sections from the manuscript (include quotes in quotation marks "like this" to indicate direct quotes from your manuscript), or elaborate on this item by providing additional information not in the ms, or briefly explain why the item is not applicable/relevant for your study

"Conflicts of Interest
JM, CF, MU, DB, and DU are current employees of Pivot Health Technologies Inc. (Pivot), the developer of the Pivot smoking cessation program. They receive salary and stock options from Pivot. DU is the President and CEO of Pivot and an investor in the company. JG is a paid statistical consultant."

About the CONSORT EHEALTH checklist

As a result of using this checklist, did you make changes in your manuscript? *

- ○ yes, major changes
- ○ yes, minor changes
- ◉ no

What were the most important changes you made as a result of using this checklist?

We mirrored this manuscript off of the first manuscript from this RCT (https://mhealth.jmir.org/2022/11/e41658/). When going through this CONSORT EHEALTH checklist process for that first manuscript, we made associated changes to the manuscript based on this process. Those changes then carried through to the current manuscript.

How much time did you spend on going through the checklist INCLUDING making changes in your manuscript *

We spent 4-5 hours going through the checklist.

As a result of using this checklist, do you think your manuscript has improved? *

- ○ yes
- ○ no
- ◉ Other: We made the changes for the first published paper from this RCT and

Would you like to become involved in the CONSORT EHEALTH group?

This would involve for example becoming involved in participating in a workshop and writing an "Explanation and Elaboration" document

- ○ yes
- ◉ no
- ○ Other:

Clear selection

Any other comments or questions on CONSORT EHEALTH

Your answer

STOP - Save this form as PDF before you click submit

To generate a record that you filled in this form, we recommend to generate a PDF of this page (on a Mac, simply select "print" and then select "print as PDF") before you submit it.

When you submit your (revised) paper to JMIR, please upload the PDF as supplementary file.

Don't worry if some text in the textboxes is cut off, as we still have the complete information in our database. Thank you!

Final step: Click submit !

Click submit so we have your answers in our database!

Submit Clear form

Never submit passwords through Google Forms.

This form was created outside of your domain. Report Abuse - Terms of Service - Privacy Policy

Google Forms
